# Tailored Supramolecular Additives to Control the Crystallization Process and Morphology of MAPbI_3_

**DOI:** 10.1002/smll.202410230

**Published:** 2025-02-05

**Authors:** Meike Kuhn, Felix A. Wenzel, Christopher Greve, Klaus Kreger, Matthias Schwartzkopf, Hans‐Werner Schmidt, Helen Grüninger, Eva M. Herzig

**Affiliations:** ^1^ Dynamics and Structure Formation – Herzig Group University of Bayreuth Universitätsstraße 30 95447 Bayreuth Germany; ^2^ Macromolecular Chemistry I and Bavarian Polymer Institute University of Bayreuth Universitätsstraße 30 95447 Bayreuth Germany; ^3^ Deutsches Elektronen‐Synchrotron DESY Notkestr. 85 22607 Hamburg Germany; ^4^ Bavarian Center for Battery Technology (BayBatt) and Inorganic Chemistry University of Bayreuth Universitätsstraße 30 95447 Bayreuth Germany

**Keywords:** additive engineering, crystal morphology, crystallization control, GIWAXS, perovskites, solid‐state NMR spectroscopy, supramolecular additive

## Abstract

Perovskite films feature unique optoelectronic properties, rendering them promising for electronic devices. The properties depend on the morphology on a broad range of length scales from nanometers to millimeters, influenced by a variety of factors. However, controlling the morphology is challenging. A tailored supramolecular additive, N, N’‐bis(2‐aminoethyl) terephthalamide is developed to control the intermediate and perovskite crystallization of methyl ammonium lead iodide (MAPbI_3_) and enhance the thermal and moisture stability in the final film. Reversible coordinative interactions of the carbonyl groups with Pb^2+^ ions via Lewis acid‐base adduct and subsequent ion–ion interactions of the peripheral ammonium groups with the perovskite grain boundaries are combined which is stabilized by a strong hydrogen bonding pattern formed between the amide moieties of the additive molecules. Adding low amounts of this additive to the precursor solution significantly decelerates the structure formation and systematically reduces the crystallite size. Slower growth of the intermediate phases and the incorporation of the additive to the grain boundary is observed with multiple time‐resolved techniques. Evidence for the formation of single‐molecule interlayers between the MAPbI_3_ crystals and the presence of directed supramolecular interaction between additive molecules is shown. Transferability of this approach to other perovskites is anticipated, paving the way to improved processing control and stability.

## Graphical abstract

A method is presented to control MAPbI₃ nanostructure during blade coating by using a tailored supramolecular additive. This additive interacts with Pb^2^⁺ ions through Lewis acid‐base adducts and attaches to perovskite grain boundaries via peripheral amines and strong hydrogen bonding. Single‐molecule interlayers form between MAPbI₃ crystals, enhancing film stability through supramolecular interactions among the additive molecules.

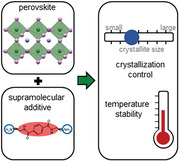

## 1 Introduction

Hybrid perovskites are highly versatile materials with various applications in devices including photovoltaics,^[^ ^1^, ^2^, ^3^, ^4^, ^5^ ^]^ light‐emitting diodes (LEDs),^[^ ^6^, ^7^, ^8^ ^]^ and photodetectors,^[^ ^9^ ^]^ where precise morphology control can dramatically influence device performance. The requirements on film uniformity,^[^ ^10^ ^]^ crystallinity,^[^ ^11^ ^]^ defect density,^[^ ^12^ ^]^ optical absorption,^[^ ^13^, ^14^, ^15^ ^]^ charge carrier transport,^[^ ^16^ ^]^ stability,^[^ ^17^ ^]^ and emission wavelength^[^ ^18^ ^]^ depend strongly on the anticipated application. Material engineering and fabrication process optimization are required to control these properties. Simple processing routines to reproducibly obtain such properties are obligatory for industrial application and upscaling, and therefore understanding and the ability to control these processes is necessary.^[^ ^19^, ^20^ ^]^

MAPbI_3_ films processed from dimethylformamide (DMF) are known to form its final structure via different stages starting from fully dissolved precursors in solution, with the subsequent evaporation‐driven intermediate phase formation and growth, followed by (partial) conversion into the final perovskite material during drying and continued conversion into the final perovskite material during thermal annealing. A successful strategy for manipulating MAPbI_3_ properties is the use of additives (e.g., ionic liquids,^[^ ^21^ ^]^ organic gelators,^[^ ^22^ ^]^ polymers,^[^ ^23^ ^]^ and bacteriophages^[^ ^24^ ^]^) in the initial solution to influence the final film structure. Recent publications have demonstrated that intermolecular forces and bondings can address a particular stage during structure formation.^[^ ^25^ ^]^ We demonstrate in this work, that additives with different, tailored chemical interactions allow access to the control of all stages during the structure formation process, leading to controlled growth and stability of the final morphology.

Targeting specific bonding mechanisms enables precise control over the structural and electronic characteristics of the perovskite film, thereby optimizing its performance and stability.^[^ ^26^ ^]^ Examples of additives enabling these interactions are Lewis acids, ammonium salts, ionic liquids, and Lewis bases. Lewis bases interact with the intermediate phase crystallites, e.g. via carbonyl and sulfur groups.^[^ ^27^, ^28^ ^]^

Molecules with amino‐functionalized groups may interact with MAPbI_3_ grain boundaries in two ways. First, if these molecules are present, a form featuring ammonium groups then can replace MA^+^ at the grain boundaries, which represents ion‐ion interaction. Secondly, molecules that have amino groups can act as Lewis bases, interacting with Lewis acid surface defects.^[^ ^26^ ^]^ After thermal annealing of the films, the additives accumulate at the grain boundaries and form a protective layer that suppresses the degradation of perovskites.^[^ ^29^, ^30^, ^31^ ^]^

However, typical amino‐functionalized additives have relatively low boiling points, which limits their ability to influence the morphology of the perovskite during the final thermal annealing step.^[^ ^32^ ^]^ Supramolecular additives have been shown to improve the process robustness and trap state passivation.^[^ ^22^, ^33^ ^]^ Here, we demonstrate that supramolecular chemistry can stabilize additives against evaporation since strong (and directed) non‐covalent interactions between additive molecules strongly reduce volatility. In this paper, we combine Lewis base interactions with ammonium functionalized groups and additional supramolecular interactions to control all stages in the structure formation process.

This study systematically examines the role of different building blocks to control and stabilize the morphology of the final MAPbI_3_ layer and presents a concept for a tailored supramolecular additive for perovskite materials. To monitor the crystallization process, we use time‐resolved in‐situ optical microscopy,^[^ ^34^ ^]^ light scattering,^[^ ^21^, ^35^, ^36^ ^]^ and photoluminescence (PL)^[^ ^21^, ^36^, ^37^, ^38^ ^]^ measurements. Applying static X‐ray scattering techniques and scanning electron microscopy (SEM), we analyze the final film morphology. With solid‐state nuclear magnetic resonance (NMR) spectroscopy, we can confirm the coordination of the additive at perovskite interfaces and supramolecular properties of the additive within the MAPbI_3_ film, which allows us to understand the differences in the thermal and moisture stability observed for the final films.

## 2 Process Design and Results

### 2.1 Molecular Design

Recently, we demonstrated the use of supramolecular additives capable of nucleating poly(3hexyl)thiophene from the polymer melt as well as from solution.^[^ ^39^, ^40^ ^]^ This additive class relies on nanostructure formation via directed strands of hydrogen bonds and a functional periphery providing an attractive and epitaxial surface for the nucleation of the polymer. Similarly, we devised and synthesized here a tailored supramolecular additive, which is able to attractively interact with the intermediate phase as well as the final perovskite structure and provides improved stabilization via hydrogen bond formation. The surface coverage of MAPbI_3_ structures with the additive therefore regulates the crystallization process of the intermediate phase and compartmentalizes the subsequent MAPbI_3_ crystallization. This limits the excessive needle growth, slows the growth in general, and hence reduces the size of the perovskite grains.

To achieve this control over the different states of MAPbI_3_ formation, we consider the following requirements: a) the supramolecular additive is designed to coordinate with the Pb^2+^ ions during the intermediate phase, b) the end groups coordinate with the converted MAPbI_3_ crystallites, anchoring the end groups on the grain boundary of the perovskite, and c) the additive is able to form directed secondary interactions between additive molecules to reduce volatility and mobility of the additive itself.

**Figure 1:**
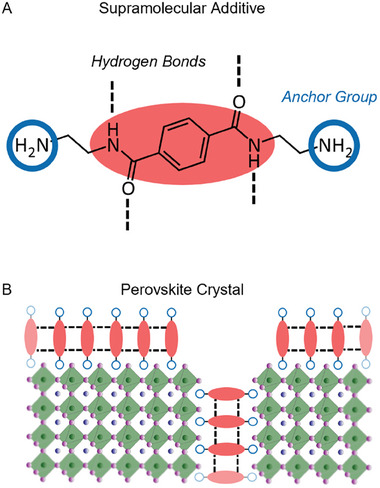
A) Chemical structures of the supramolecular additive N,N’‐bis(2‐aminoethyl) terephthalamide. The molecular design combines strong supramolecular interactions of amide groups and the ability to attach to perovskite crystals due to the terminal amine moieties. B) Conceptual illustration for the supramolecular additive's interaction with the MAPbI_3_ by forming stable mono interlayers between perovskite crystals.

The molecular design comprises benzene 1,4‐biscarboxamides as the core structure, suitable to form the linear hydrogen pattern and to interact with the Pb^2+^ ions. The core structure is linked to peripheral functional 2‐aminoethylene moieties, which can interact with the perovskite crystallites replacing the methylammonium species during the perovskite conversion. Therefore, the supramolecular additive N,N’‐bis(2‐aminoethyl) terephthalamide is devised, in short **S**upramolecular C2‐symmetric **B**enzene 1,4‐**B**iscarbox**A**mide (SBBA) (the structure is shown in **Figure** **1**A).

In more detail (as shown in Figure 1B), we expect the carbonyl group (C═O) of the amides to interact with the Pb^2+^ during the intermediate phase formation via a strong coordinative interaction between the C═O and the Pb^2+^ as found for additives with a similar binding motif.^[^ ^27^, ^41^, ^42^ ^]^ Thereby, a Lewis acid‐base adduct can be formed.^[^ ^27^ ^]^ We expect the oxygen of the carbonyl group (Lewis base) to interact with the Pb^2+^ (Lewis acid) via a secondary, reversible Pb⋯O═C interaction.

The terminal amine moieties are protonated in solution yielding an ammonium group. During perovskite conversion and in the final film the resulting ammonium groups can coordinate with the MAPbI_3_ interfaces via ionic interactions with methylammonium vacancies. The ethylene spacer adds flexibility to the SBBA, allowing the protonated amines to reach the desired position.

Finally, the amide groups, which are C2‐symmetrically linked to the central benzene core, form two strands of directed hydrogen bonds between the adjacent additive molecules, resulting in stable supramolecular structures.

The SBBA was synthesized using a straightforward amidation reaction of terephthalic acid dimethyl ester with a large excess of ethylenediamine. Details on the synthetic route, the synthetic procedure, and the molecular characterization are given in Section S1 (Supporting Information). The SBBA features a high melting point of ≈180 °C, preventing volatility even at elevated temperatures of ≈100 °C. Due to the polar character, the SBBA is soluble in DMF. The non‐volatile character and the good solubility of the SBBA allow straightforward processing as commonly used in a one‐step procedure by adding the additive directly to a mixture of the precursors methylammonium iodide and lead iodide in DMF. Consequently, the SBBA is easily employed in established processing routes.

### 2.2 Intermediate Phase Formation

To evaluate the effect of the SBBA on the film formation process of MAPbI_3_, solutions with different concentrations of SBBA in DMF were prepared. For all concentrations, a single‐step blade‐coating process at 40 °C substrate temperature is used. For these processing conditions, the crystallization path of the MAPbI_3_ proceeds via an intermediate phase. In this stage, intermediate phases form due to a strong interaction between the precursors and the polar solvent molecules of DMF, resulting in the formation of (MA)_2_(DMF)_2_Pb_2_I_6_ intermediate states.^[^ ^19^, ^43^, ^44^ ^]^

**Figure 2:**
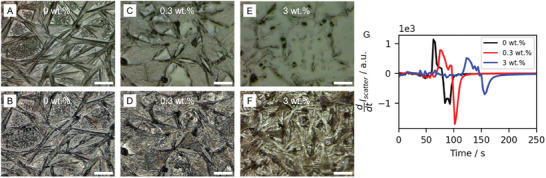
A–F) Optical microscopy images in reflection mode at different times during the drying of the films for different weight fractions of SBBA. 10 s after the optically observable crystallization onset (top row) and the final pre‐annealed film (bottom row). The scale bar is 100 µm. G) Time derivative of the intensity of the light scattering at 2.59 eV (extracted from Figure S3, Supporting Information) in seconds after film coating.

The occurrence of the intermediate phase formation is tracked using time‐resolved optical microscopy and light scattering. Exemplary optical microscopy (OM) images of the film formation are depicted in **Figure** **2**A–F for 0 wt.%, 0.3 wt.%, and 3 wt.% SBBA after 10 s of the optically observable crystallization onset (wet, top image in each column) and the final pre‐annealed film (bottom image in each column). From the OM time series, we observe intermediate phase formation and growth in all samples, followed by a color change, providing evidence for the successful conversion into MAPbI_3_. We find that the SBBA drastically influences the duration of the intermediate stage. The reference sample with 0 wt.% of SBBA shows the formation of the classical elongated needle‐like structure on the micron scale, consisting of intermediate states and already converted perovskite grains. There are significant cavities between the individual needles, which are partially filled with smaller structures grown in a secondary process. The needle‐like shape results from the formation of the (MA)_2_(DMF)_2_Pb_2_I_6_ intermediate states during the film drying, resulting in growth along one axis.^[^ ^44^ ^]^ The second sample with 0.3 wt.% of SBBA features slightly shorter but broader needle‐like structures, resulting in smaller cavities between the needles. The final sample with 3 wt.% of SBBA shows even shorter needle‐like structures, and cavities can no longer be identified using optical microscopy. Detailed time‐resolved analysis of the OM images (see Section S2, Supporting Information) can quantify the differences in the intermediate phase formation rate and directionality of the macroscale structures. In the solution with 0 wt.%, elongated intermediate structures form at a rate of ≈60 µm per second along the long axis. In contrast, in the sample with 3 wt.% SBBA intermediate structures form with a rate of less than 20 µm per second and are observed to grow into additional directions. Hence SBBA reduces the growth rate of the (MA)_2_(DMF)_2_Pb_2_I_6_ intermediate states and changes the directionality of the growth.

To characterize the intermediate phase formation in more detail, light scattering from a blue LED signal is used (Figure S3, Supporting Information).^[^ ^36^ ^]^ Light scattering takes place if objects in the drying solution are larger than ≈0.465 µm.^[^ ^36^, ^45^ ^]^ The increase in light scattering signal can, therefore, be associated with the formation of intermediate states.^[^ ^19^, ^36^, ^43^, ^44^ ^]^ The time derivative of the light scattering intensity at 2.59 eV is shown in Figure 2G. The initial positive peaks in that graph correlate to the increase in light scattering due to the formation of intermediate states, while the subsequent negative peaks of these curves indicate the reduction in light scattering and, therefore, the conversion into MAPbI_3_.^[^ ^36^ ^]^ The position of the positive peak corresponds to the point in time with the highest formation rate of intermediate states and the width to the duration of the intermediate phase formation. The graph shows that the light scattering due to intermediate phase formation is delayed and gets broader with the addition of SBBA.

In summary, the addition of SBBA to the precursor solution impacts the growth mechanism of the intermediate phase in two ways. First, the onset of intermediate phase formation is delayed, and second, the growth process is slowed down on a broad range of length scales. The intermediates from the solution without SBBA have the typical growth along one axis, while the intermediates that grow more slowly from the solution show increased growth in additional directions with increasing SBBA concentrations.

We suggest that reversible Lewis acid‐base adduct^[^ ^27^ ^]^ between the carbonyl groups of the SBBA (Lewis base) and Pb^2+^ ions (Lewis acid) occur and are responsible for these changes in the intermediate stage.

### 2.3 Intermediate Phase Formation with Reference Structures

**Figure 3:**
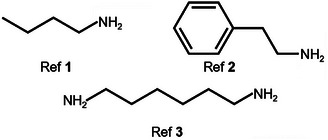
Molecular structures of the used reference compounds. Butylamine (ref. material 1), phenylethylamine (ref. material 2), and hexane‐1,6‐diamine (ref. material 3) feature terminal amine end groups like the SBBA additive. However, in contrast to SBBA, amide groups are absent in these small molecules and, therefore, they cannot form a pattern of strong and directed hydrogen bonds.

To identify which interactions between SBBA and Pb^2+^ ions are influencing the intermediate phase formation, time‐resolved light scattering experiments are repeated using three different reference compounds (see **Figure** **3**; Section S4 and Figure S8, Supporting Information). All reference compounds have terminal aliphatic amine groups, but the amide groups were omitted. Since these compounds do not have a carbonyl group, the formation of a Pb⋯O═C coordinative bond is not possible. For none of the reference structures, the width of the initial peak of the time derivative data is broadened (Figure S8, Supporting Information), thus no increase in the duration of intermediate state formation occurs. Hence, the prolonged duration of the intermediate phase formation can be attributed to the carbonyl groups of the additive. Interestingly, the diamino‐functionalized additive (ref. material 3 (hexamethylenediamine)) shows a delay in the onset of the intermediate phase formation, like SBBA, but no slowing down of the formation process. Therefore, we conclude that the Pb⋯O═C interaction is not solely responsible for the delay in the onset of the intermediate phase formation. Only the prolonged duration can be directly linked to the presence of the carbonyl groups.

### 2.4 Perovskite Formation

**Figure 4:**
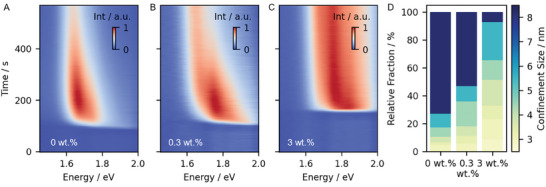
Time‐resolved photoluminescence spectroscopy of MAPbI_3_ with A) 0 wt.%, B) 0.3 wt.%, or C) 3 wt.% SBBA in DMF. The MAPbI_3_ crystals give rise to the PL signal, which shifts to lower energies upon crystal growth. With the additive PL at higher energies is observed, indicating smaller crystallite sizes. The intensity scale bar is normalized for better comparison. D) Calculated fraction of crystallite sizes for the films prior annealing for different additive concentrations.

The conversion of the intermediate phase into the actual perovskite material is characterized using the second part of the time‐resolved light scattering and photoluminescence (PL) measurements. The delayed occurrence of the negative peak of the curve in Figure 2G shows that the onset of the conversion process is delayed when SBBA is added to the solution. A PL signal is detectable as soon as MAPbI_3_ is formed. **Figure** **4**A–C shows the temporal evolution of the PL signal for 0 wt.%, 0.3 wt.%, and 3 wt.% SBBA. Initially, while all precursors are dissolved in the solution and subsequently while the intermediate phase is formed, no photoluminescence signal is observed. Upon further solvent evaporation, the intermediate states start to convert into perovskite crystals, giving rise to an initial PL signal. For the reference sample (0 wt.%), a distinctive shift of the PL signal toward lower energies is observed, which corresponds to perovskite crystal growth.^[^ ^18^, ^36^, ^46^, ^47^ ^]^

The sample with 0.3 wt.% shows a significantly broader PL spectrum as compared to 0 wt.%. It has been shown that quantum confinement of perovskite crystallites results in distinct PL peak positions that correlate with confinement size when the energy is higher than the typical PL bulk energy of 1.6 eV.^[^ ^21^, ^36^ ^]^ The PL data can therefore be analyzed with respect to intensity contributions at different energies. Hence, the 0.3 wt.% sample already indicates that the size distribution of perovskite grains is shifted to smaller grains, particularly in the early stage of the conversion in comparison to the neat sample. The sample with 3 wt.% of SBBA shows a much broader PL spectrum from 1.63 to 1.9 eV, consisting of PL contributions that are stronger at higher energies than for lower SBBA content. Further, these high energy contributions remain during the entire drying process. This is evidence that SBBA induces a much broader distribution of confinement sizes and a significantly increased fraction of small crystallites.

The PL signal is therefore analyzed in detail (Section S3, Supporting Information) to extract the confinement size distribution which is obtained by fitting each spectrum using multiple hyperbolic secants. The distribution of confinement sizes is extracted for each timeframe using the center of the functions and the relative PL intensity of each peak. We observe, that without additives, the larger confinement sizes grow continuously, but with SBBA, there is an increased fraction of smaller confined sizes within the film (Figure S6, Supporting Information). Hence, the overall size distribution is shifted to smaller sizes with the SBBA (see Figure 4D). Thus, we conclude that the MAPbI_3_ crystallite growth must be restricted when using the SBBA.

After the blade coating, the samples are annealed at 100 °C for 10 min and the static PL is measured afterward at room temperature (Figure S7, Supporting Information). As expected during thermal annealing with its associated grain growth, the PL peak shifts to lower energies for all samples. However, the higher energy PL signal is still present for sufficient SBBA content, while it disappears if no or low amounts of SBBA are added. This demonstrates that the crystallite growth is hindered, suggesting that the SBBA is still present at the interfaces after thermal annealing. It is worth noting that the sample with 3 wt.% of SBBA features significantly higher intensity of the PL than the other samples. The increase in the PL signal hints at an improved quality of the material and fewer defects or trap states in the film, as these promote non‐radiative decay.

### 2.5 Perovskite Formation with Reference Structures

To identify the reason for the broadening of the confinement size distribution and its stability, we again utilize our reference additives that lack amide groups but feature one or two terminal amine groups (Figure 3). The additives were added to the precursor solution, and the crystallization process was subsequently analyzed in the same way as described in the previous sections. The corresponding figures are shown in Figures S8–S11 (Supporting Information).

Time‐resolved PL studies on the samples with reference additives with one amine end group (ref. material 1: butylamine and ref. material 2: phenylethylamine) reveal no changes in the perovskite conversion process and the final confinement size distribution is comparable to the neat MAPbI_3_ films. However, using ref. material 3 (hexamethylenediamine) with two amine end groups does significantly broaden the confinement size distribution during the perovskite conversion process like the SBBA, although there is no central chemical moiety, responsible for directed supramolecular interactions (Figures S9–S10, Supporting Information). Therefore, double amine end groups seem essential for efficient confinement size reduction in the dry, pre‐annealed film. However, the PL signals of all reference samples shift to lower energies after thermal annealing, indicating unhindered perovskite grain growth during heating, as observed for the sample without SBBA (Figure S11, Supporting Information). Although ref. material 3 initially confines the perovskite crystallites, this confinement is significantly lost during thermal annealing, while SBBA maintains this confinement due to the intramolecular interactions between the additive molecules.

Thus, while the reference structures with two amine end groups can influence the final pre‐annealed perovskite confinement size, only the ionic interaction between the perovskite and ammonium end group is insufficient to inhibit crystallite fusion during annealing. However, the additional supramolecular interactions, i.e., the hydrogen bond pattern, present for the SBBA lend the system sufficient stability to inhibit crystallite fusion during thermal annealing.

Overall, the comparison of the SBBA with the reference structures shows that all chemical design elements of the SBBA are essential for guiding the structure formation process throughout all stages. The carbonyl group is responsible for the interaction of the SBBA with the Pb^2+^ during the intermediate phase formation. The two amine end groups, which protonate in the solution, interact with the newly formed perovskite during the conversion process, and the central moiety containing amides is necessary for the supramolecular interactions, which are fundamental to ensure stability during the thermal annealing process.

### 2.6 Final Film Properties

**Figure 5:**
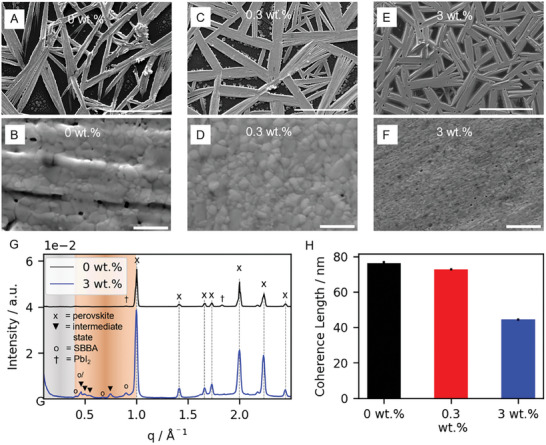
SEM measurements of the annealed MAPbI_3_ film with A,B) 0 wt.%, C,D) 0.3 wt.%, and E,F) 3 wt.% of SBBA. The scale bar for A,C,E) is 40 µm and for B,D,F) 1 µm. G) Radially averaged WAXS data of sample powder with 0 wt.% (black) and 3 wt.% additive (blue). The colored ranges indicate the typical ranges for 2D perovskite signals (grey) and intermediate phases (orange). H) Calculated coherence length of MAPbI_3_ films with 0 wt.% (black), 0.3 wt.% (red), and 3 wt.% (blue) of SBBA from XRD measurements.

The SEM images in **Figure** **5**A–F shows thermally annealed MAPbI_3_ films with different concentrations of SBBA at two different magnifications each. The perovskite structures for all films show a needle‐like shape on the macro‐scale. With increasing amounts of SBBA, the needles become shorter and exhibit fewer cavities. Secondly, the needle‐like morphology becomes broader and denser. This alteration in shape is in line with the observations during the intermediate phase growth in OM. The larger magnification of the samples with 0 wt.% and 0.3 wt.% SBBA show the typical broad distribution of grain sizes of MAPbI_3_ processed from DMF.^[^ ^48^ ^]^ In comparison, significantly smaller, elongated structures are observed on the nanoscale for the sample with 3 wt.% SBBA. This is in accordance with the observation from the PL measurements that crystallite fusion is suppressed during the thermal annealing step for sufficient SBBA content. This indicates again that the SBBA remains within the sample despite thermal annealing.

The crystal structure of the final annealed MAPbI_3_ film is analyzed using X‐ray scattering. Wide‐angle X‐ray scattering (WAXS) measurements show that the MAPbI_3_ exhibits the typical tetragonal structure at room temperature independent of the SBBA content, as shown in Figure 5G. For the sample with SBBA, additional peaks occur in the region between 0.4 and 1.0 Å^−1^ (orange shaded range in Figure 5G) These additional features are assigned to the neat SBBA and the (MA)_2_(DMF)_2_Pb_2_I_6_ intermediate states^[^ ^49^ ^]^ within the sample (see Figure S12A–C and Table S2, Supporting Information). The latter observation shows that despite the annealing at 100 °C for 10 min, solvent is still present in the sample. Furthermore, it is confirmed that no layered 2D perovskites are formed in the presence of the SBBA, also supported by static UV/Vis absorption, as this shows no evidence of traditional 2D perovskite structures^[^ ^50^, ^51^, ^52^ ^]^ (see Figure S12D, Supporting Information). We further note that the sample without SBBA shows a small but distinct peak that we can assign to PbI_2_, implying either incomplete perovskite formation or degradation. In contrast, the sample with SBBA shows no PbI_2_ reflex.

The average coherence length in vertical film direction is studied by X‐ray diffraction (XRD) of the samples (see Figure S13, Supporting Information). The (110) MAPbI_3_ peak is analyzed using the Scherrer analysis to extract the coherence length of the MAPbI_3_ crystallites. The crystallites in the sample without SBBA have an average size of (76.4 ± 1.2) nm. The addition of 0.3 wt.% of the SBBA slightly reduces this size to (72.8 ± 1.1) nm. However, the sample with 3 wt.% of SBBA shows a reduction of more than 40% down to (44.5 ± 0.4) nm (see Figure 5H).

Overall, we observe that the SBBA hinders the crystallite fusion during the thermal annealing process at 100 °C, maintaining the smaller nanostructures, showing that we can successfully tune the final grain size in the perovskite film beyond temperature annealing with varying SBBA concentrations.

### 2.7 Solid‐State NMR Spectroscopy

**Figure 6:**
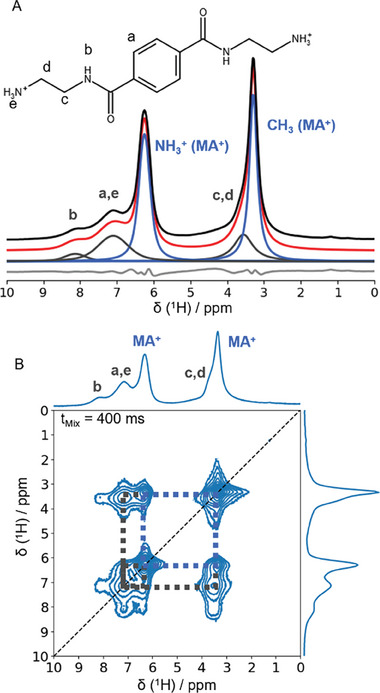
A) Quantitative 1D ^1^H MAS NMR spectrum and B) 2D ^1^H ^1^H EXSY NMR spectrum of MAPbI_3_ with 3 wt.% of SBBA. A) The deconvolution of the experimental NMR spectrum (black) is shown in blue for the ^1^H signals arising from MA^+^ cations (NH_3_^+^, CH_3_) in the MAPbI_3_ structure and in dark grey for protons of the additive (assignment a–e), the sum is indicated in red. The difference between deconvolution and experimental spectrum is depicted in light grey. B) Correlations between ^1^H signals of the MAPbI_3_ are indicated in blue, while correlations between ^1^H signals of the additive and the perovskite are indicated by the dark grey squares.

To understand the interaction of the SBBA with the MAPbI_3_ crystallites on a molecular level, we conduct solid‐state NMR spectroscopy of MAPbI_3_ with 3 wt.% of SBBA. The quantitative ^1^H MAS NMR spectrum **Figure** **6**A displays five distinguishable ^1^H signals (Table S3, Supporting Information), of which the sharper resonances at 3.3 and 6.3 ppm are readily assigned to the NH_3_^+^ and the CH_3_ species of the methylammonium (MA^+^) cations in the perovskite.^[^ ^53^, ^54^ ^]^ For the assignment of the remaining three signals to the different ^1^H species of the SBBA, we use 2D ^1^H‐^14^N HMQC NMR and ^1^H‐^1^H DQSQ NMR spectra (Figure S14, Supporting Information). In the former, we observe the ^1^H‐^14^N correlation of the NH_3_^+^ species of the MA^+^, as well as a correlation between the ^1^H signal at 7.1 ppm and a ^14^N signal at ‐379 ppm. Both, the ^14^N chemical shift and its small half width, which are very close to the ones observed for MA^+^ (δ (^14^N) = −415 ppm), show that the amine moiety of the SBBA is protonated to its ammonium form within the MAPbI_3_ film. The ^1^H‐^1^H DQSQ NMR spectrum aids in assigning the signal at 8.1 ppm to the NH groups of the amides, as this is the signal with solely cross‐correlations (off‐diagonal) and therefore is just near ^1^H species with different chemical environments. The aromatic protons CH_Ar_ of the benzene are expected at chemical shifts between 6 and 8 ppm and thus are also assigned to the signal at 7.1 ppm, while CH_2_ groups typically possess shifts <4 ppm and thus cause the remaining signal at 3.6 ppm. This assignment is also corroborated by the signal integrals in the 1D ^1^H MAS NMR spectrum being 2:10:8 according to 2 × NH:2 × NH_3_^+^ + 4 × CH_Ar_:4 × CH_2_.

In order to show whether the SBBA is interacting with the MAPbI_3_, we recorded 2D ^1^H ^1^H exchange (EXSY) NMR spectra (Figure 6B) and Figure S15, Supporting Information). The 2D spectrum shows cross‐correlations between ^1^H signal at 7.1 ppm corresponding to aromatic and ammonium groups of the SBBA and the ^1^H signals of the perovskite at 3.3 and 6.3 ppm (Figure 6B, grey dashed squares). These cross‐correlation signals reveal that the SBBA molecules interact (dipolar coupled) with the MA cations within the perovskite lattice due to a close spatial proximity. Since the SBBA‐perovskite cross‐correlation signals are only observed for the ^1^H species at 7.1 ppm, this indicates that the ammonium groups of the SBBA coordinate to the MAPbI_3_ interfaces and grain boundaries and thus act as the intended “anchor groups”.

The ^1^H NMR spectra further can be used to characterize the distribution and self‐interaction of the SBBA within the sample. The fact that solely a single ^14^N chemical shift is probed in addition to a single ^1^H shift of the NH_3_^+^ SBBA species suggests that all SBBA amine groups are protonated during synthesis and all end groups are present in a similar environment, implying that single SBBA layers are formed between perovskite crystallites engaging with both ends with MAPbI_3_. Furthermore, we observe similar ^1^H chemical shifts for the NH groups of the neat SBBA and the SBBA in the MAPbI_3_ (Figure S16 and Table S4, Supporting Information) showing that similar environments are present for both cases. Especially, varying hydrogen bonding situations lead to significant changes in ^1^H chemical shifts.^[^ ^55^, ^56^ ^]^ As the change in ^1^H shift is as small as 0.3 ppm, we expect that also the hydrogen bonding situation is rather similar for the neat SBBA and the SBBA at the MAPbI_3_ interfaces. As such, the intermolecular hydrogen bonds between the SBBA molecules are preserved, validating that the anticipated supramolecular structure of the SBBA is formed. Hence NMR measurements show that a) SBBA is attaching to the perovskite interface, b) SBBA is only observed at perovskite interfaces and not as SBBA bulk material, and c) hydrogen bond strands are formed within these interfacial layers.

### 2.8 Stability

**Figure 7:**
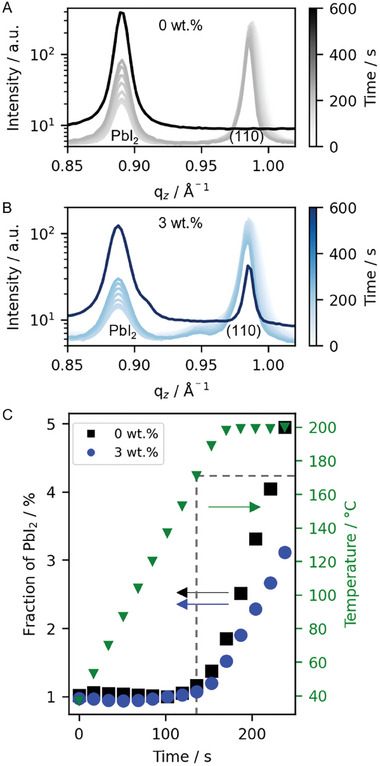
Degradation of perovskite film with A) 0 wt.% and B) 3wt.% of SBBA. Gradually changing colors show initial degradation and solid lines the final degraded state after 600 s at a new film position without beam damage. C) Initial extracted decomposition curve of the MAPbI_3_ by tracking the fraction of the $\frac{PbI_{2}}{\left(PbI_{2}+MAPbI_{3(110)}\right)}$ content.

Since grain boundaries are used for ion migration during thermally‐induced degradation^[^ ^57^ ^]^ and for the formation of PbI_2_‐water complexes during water‐induced degradation,^[^ ^57^, ^58^ ^]^ we examine the supramolecular stabilized, passivating properties of SBBA for different temperature and humidity regimes. First, we conduct a thermally accelerated aging study by storing samples of MAPbI_3_ with 0 wt.%, 0.3 wt.%, and 3 wt.% of the SBBA at 80 °C in air with a humidity of (35 ± 3) % and optically examining the color change (see Figure S17, Supporting Information). While the freshly thermally annealed MAPbI_3_ films display a solid dark color for all samples, the degradation product lead(II) iodide (PbI_2_) appears yellow. While samples with low SBBA content degrade quickly, the 3 wt.% SBBA sample shows much less degradation as indicated by the colors. Hence, this aging study clearly shows suppression of decomposition with an increased amount of SBBA despite the significantly increased amount of grain boundary area. To investigate the degradation evolution in a more detailed manner, we carried out an accelerated thermal aging procedure in nitrogen to examine the onset of temperature‐induced degradation and resulting decomposition of the films using in situ GIWAXS. Here, we investigate the samples during heating up to 200 °C with 1 °C s^−1^, followed by holding the temperature at the final temperature for 5 min to induce rapid degradation. **Figure** **7**A,B displays the vertical cake cuts of the 2D GIWAXS patterns showing the (001) PbI_2_ and (110) MAPbI_3_ peak as a function of time for the samples with 0 wt.% and 3 wt.% SBBA. Initially, during the heating process, only a change in the position of the (110) perovskite peak is observed, indicating thermal expansion. After 140 s, when reaching 170 °C, the intensity of the PbI_2_ peak increases while the intensity of the perovskite peak decreases, indicating the onset of degradation of MAPbI_3_. While the neat sample fully degrades during the thermally accelerated aging procedure, as indicated by the disappearance of the (110) peak (Figure 7A; Figure S18, Supporting Information), the sample with 3 wt.% of SBBA shows a suppressed degradation with a remaining reflex attributed to (110) MAPbI_3_ peak (see Figure 7B; Figure S18, Supporting Information). This suppression is also observed when analyzing the PbI_2_ conversion with time (see Figure 7C). Overall, the effect of the SBBA on stability was examined using two different approaches: a qualitative study to assess long‐term stability and a quantitative study under accelerated aging. Both methods demonstrate increased stability. This suggests that the coordination of the SBBA with the perovskite grain boundary and stabilization via supramolecular interactions seems to suppress ion migration, thereby significantly enhancing the stability of the perovskite films. It has been shown in literature that surface passivation and increased material stability are often linked to enhanced optoelectronic properties. This relationship is crucial for improving the performance of perovskite materials, as defects on the surface can serve as trap states that hinder charge transport and reduce efficiency.^[^ ^24^, ^59^ ^]^ The passivation of such surface defects, often achieved through the incorporation of additives, not only improves the stability of the material but can also lead to more efficient charge carrier dynamics, thereby enhancing the optoelectronic properties of the material. In our study, we observed that the passivation at the interfaces significantly improved the stability of our samples. This result is consistent with previous reports, where passivated surfaces also showed higher stability. Also, more favorable electronic characteristics, such as increased photogenerated charge carrier lifetimes and improved conductivity have been observed.^[^ ^23^ ^]^

## 3 Conclusion

We presented a tailored supramolecular benzene 1,4‐biscarboxamide (SBBA) additive to systematically interact with the intermediate states and the MAPbI_3_ interfaces during each stage of the film formation. We demonstrated with time‐resolved characterization of the film formation and static measurements that the SBBA slows down the crystallization of the intermediate phase, delays the perovskite conversion, and reduces the final crystallite sizes. With NMR, we confirm that the SBBA is coordinated to the formed perovskite crystallites in a single layer while forming stabilizing strands of hydrogen bonds. The SBBA, therefore, successfully attaches to the perovskite grain boundaries, inhibiting structural growth. This surface interactive behavior is also observed for a diamino‐functionalized reference, (ref. material 3) but only the SBBA with additional directed supramolecular interactions shows significant thermal stability. This design principle is not limited to the model system MAPbI_3_ and we expect that it can be transferred to other hybrid halide perovskite material systems when adhering to the following design requirements for the SBBA or new additives in combination with suitable perovskite compositions: a) attractive, reversible interactions between additive and intermediate phase and attractive interactions with the A‐site vacancies; b) supramolecular interactions between the additive molecules in the final film; c) fulfilling size limitations between crystal unit cells and hydrogen bond length. We expect this approach to provide new opportunities for precise structural control.

## 4 Experimental Section

### Precursor Solutions

Materials for films of MAPbI_3_ were used as received. Methylammonium iodide was bought from Greatcell Solar and Lead(II) iodide (99,99% trace metal basis) was obtained from Tokyo Chemical Industry. The precursors were dissolved in DMF (99,8%, Acros Organics) with a concentration of 0.778 m. To get the final concentration of 0.7 m the corresponding amount of either pure DMF or the molecular additive dissolved in DMF was added.

### Film Fabrication

Films of MAPbI_3_ were produced on glass substrates by blade coating in a one‐step process. The glass substrates were cleaned in an ultrasonic bath with soap water (Alconox), deionized water, ethanol, acetone, and isopropanol and treated with oxygen plasma before use. The prepared MAPbI_3_ precursor solution was used to blade coat films with a home‐built setup under an ambient atmosphere on the prepared glass substrates.^[^ ^60^ ^]^ The parameters were chosen as follows: 150 µm gap distance between blade and substrate, 10 mm s^−1^ coating speed, 10 mm s^−2^ initial coating acceleration, and 40 °C substrate bed temperature. After a rest time of ≈10 min after the finished slot‐die coating process, the films were transferred to a heat plate and annealed for 10 min at 100 °C in air.

### Time‐Resolved Microscopy and Spectroscopy

During the rest time (includes the wet film, the drying film, and the dried film), the in situ measurements were carried out by positioning either a time‐resolved digital microscope (Keyence VHX 970F with VH‐Z250L objective) or a custom‐made spectroscopy attachment next to the blade as shown in Figure S1 (Supporting Information). The custom‐made spectroscopy attachment was used to measure the PL spectra. An LED emitting at 465 nm in combination with focusing optics was used to excite a PL signal. Furthermore, the reflection of the LED signal could be used as a monitor for the amount of scattered light whose magnitude is increased if scattering centers are formed in the solution leading to the scattering of blue light. The PL signal was collected with an optical fiber placed over the sample. This optical fiber is attached to a spectrometer (Avantes AvaSpec‐HSC‐TEC), which records the PL spectra.

### SEM

The samples were sputtered with 1.4 nm platinum using a Cressington 208HR sputter coater and the film morphology was characterized by SEM using a Zeiss Leo 1530 instrument FE‐SEM with Schottky‐field‐emission cathode, In‐lens detector, and SE2 detector.

### WAXS

To measure WAXS, the perovskite films were scraped off the glass substrate and placed in between two Kapton foils. The neat SBBA and PbI_2_ were measured as powder. The measurements were performed in vacuum at RT on a laboratory system (Xeuss 3.0, Xenocs SAS, Grenoble, France) with a Cu K_α_ source (λ  = 1.54 Å), a Dectris EIGER2 R 1 m detector, and three different sample‐to‐detector distances of 72, 300, and 1800 mm to collect the SAXS and WAXS region. The presented *q*‐profiles were integrated over the whole azimuthal angle from 0−360°.

### XRD

The XRD patterns of the final films were recorded on a Bragg‐Brentano‐type diffractometer (Empyrean, Malvern Panalytical BV, Netherlands) equipped with a rotating spinner and a PIXcel‐1D detector using Cu‐K_α_ radiation (λ = 1.54 Å). Measurements were recorded under ambient conditions with a slit width of ¼’.

### GIWAXS

The GIWAXS data were taken with a LAMBDA 9 M detector (X‐Spectrum, Germany) at a wavelength of 1.048 Å, a sample‐detector distance of 427.55 mm, and an incident angle of 0.4° at the P03 MiNaXS^[^ ^61^ ^]^ beamline at PETRA III DESY (Hamburg, Germany). The in situ investigations were performed with a DHS1100 (Anton‐Paar, Austria) heater in a nitrogen atmosphere. The sample‐detector distance was corrected to eliminate the height change due to heat expansion of the stage and the substrate while heating. The height change was calibrated using the PbI_2_ peak, as we would expect no thermal expansion of this peak.^[^ ^32^ ^]^ The displayed GIWAXS line cuts were obtained by cake cuts along q_z_ in between 70−90°.

### Absorption

Absorption measurements of annealed samples in transmission were performed with a Jasco V670 using a 60 mm integrating sphere (ISN‐723 UV‐Visible‐NIR).

### Solid‐State NMR Spectroscopy

Solid‐state NMR experiments were performed using a Bruker Avance III spectrometer operating at a magnetic field strength of 14.1 T, corresponding to a ^1^H Larmor frequency of 600.1 MHz and ^14^N Larmor frequency of 43.4 MHz. All experiments were carried out using a Bruker 1.3 mm double resonance MAS NMR probe at a MAS frequency of 62.5 kHz unless otherwise stated. All ^1^H spectra were referenced with respect to TMS using adamantane (1.85 ppm) as a secondary reference at room temperature. For ^14^N glycine was used as a secondary reference.

High‐resolution 1D ^1^H MAS single‐pulse (SP) NMR spectra were recorded after a 90° pulse of 1.35 µs (B_1_ field strength ≈185 kHz) and a recycle delay of 60 s for SBBA@MAPbI_3_ and neat SBBA. The recycle delays were optimized for each sample to ensure quantitative NMR spectra. The ^1^H MAS NMR spectra were deconvoluted using the software ssNake.^[^ ^62^ ^]^

2D single‐quantum‐single‐quantum noesy‐type spin diffusion ^1^H‐^1^H correlation spectra^[^ ^63^ ^]^ were acquired using a ^1^H B_1_ field strength of ≈185 kHz and a mixing time of 400 ms.

2D double‐quantum‐single‐quantum (DQ‐SQ) ^1^H‐^1^H correlation spectra were acquired using the R12_2_^5^ symmetry‐based recoupling sequence^[^ ^64^ ^]^ with a 180° pulse as the basic R element. The DQ excitation time was set to 128 µs.

The 2D ^1^H‐^14^N dipolar multiple quantum coherence (HMQC) spectrum was recorded at 50 kHz spinning frequency employing a complete SR4_1_^2^ symmetry^[^ ^65^ ^]^ for the transfer of coherence order from ^1^H to ^14^N spins.^[^ ^66^ ^]^ The two ^14^N pulses were set to 16.0 µs. A rotor‐synchronized t_1_ evolution was employed between two ^14^N pulses.

## Conflict of Interest

The authors declare no conflict of interest.

## Supporting information



Supporting Information

## Data Availability

The data that support the findings of this study are available from the corresponding author upon reasonable request.
