# Supplementary material for: Tailored Supramolecular Additives to Control the Crystallization Process and Morphology of MAPbI3
Source: Small. 2025 Feb 5;21(9):2410230. doi: 10.1002/smll.202410230 (PMC11878263; doi:10.1002/smll.202410230)
Supplement: Supplementary file 1 — Supporting Information [file SMLL-21-2410230-s001.docx]

Supporting Information

Tailored Supramolecular Additives to Control the Crystallization Process and Morphology of MAPbI_3_

Meike Kuhn, Felix A. Wenzel, Christopher Greve, Klaus Kreger, Matthias Schwartzkopf, Hans-Werner Schmidt, Helen Grüninger ^*^, Eva M. Herzig ^*^

Meike Kuhn, Christopher Greve, Eva M. Herzig

Dynamics and Structure Formation – Herzig Group

University of Bayreuth

Universitätsstraße 30, 95447 Bayreuth, Germany
E-mail: eva.herzig@uni-bayreuth.de

Felix A. Wenzel, Klaus Kreger, Hans-Werner Schmidt

Macromolecular Chemistry I and Bavarian Polymer Institute

University of Bayreuth

Universitätsstraße 30, 95447 Bayreuth, Germany

Matthias Schwartzkopf

Deutsches Elektronen-Synchrotron DESY

Notkestr. 85, 22607 Hamburg, Germany

Helen Grüninger

Bavarian Center of Battery Technology (BayBatt) and Inorganic Chemistry

University of Bayreuth

Universitätsstraße 30, 95447 Bayreuth, Germany

**S1: Synthetic procedures and characterization of N,N’-bis(2-aminoethyl) terephthalamide**

*Molecular characterization*

^1^H-NMR spectra in solution were recorded on a Bruker Avance Ultrashield 300 (300 MHz) at room
temperature. For the preparation of the NMR samples, approx. 5 mg of the N,N’-bis(2-aminoethyl) terephthalamide were dissolved in 1.2 mL of D_2_O.

High resolution mass spectra (HRMS) were performed on a Finnigan MAT 8500spectrometer from Thermo-Fisher Scientific using electron spray ionization.

Infrared (IR) spectra were carried out on a PerkinElmer Spectrum 100 FT-IR spectrometer in attenuated total reflection (ATR) mode in the range from 4000 cm^-1^ to 650 cm^-1^.

The melting point (Mp) of the N,N’-bis(2-aminoethyl) terephthalamide was determined by differential scanning calorimetry using a Mettler Toledo DSC2. Approx. 10 mg of the solid were filled in crucibles. Spectra were recorded with a heating/cooling rate of 10 K·min^-1^ in a temperature range from 30 °C to 230 °C.

*Synthetic procedure of N,N’-bis(2-aminoethyl) terephthalamide (CAS: 17197-13-6)*

The synthesis of the N,N’-bis(2-aminoethyl) terephthalamide was performed in a similar manner as described in literature.^1^ 1.5 g of terephthalic acid dimethyl ester and 40 ml of dried methanol were placed in a Schlenk flask. Under argon and ice-cooling, 129.1 ml of ethylene diamine was added dropwise. The mixture was stirred at 0 °C for 30 minutes and subsequently stirred over night at room temperature. The solvents were removed under reduced pressure. To the whitish slurry 20 ml of methanol and 100 ml of toluene were added and removed thrice. The product was dried at 60 °C under reduced pressure, yielding 1.8 g (93%) of a whitish powder.

^1^H-NMR (D_2_O, 𝛿 in ppm): 2.80 (4H, t, (CO)CH_2_-), 3.42 (4H, t, (NH_2_)CH_2_-), 7.78 (4H, s, Ar).

HRMS (ESI) m/z: [M + H]^+^ calculated for C_12_H_19_O_2_N_4_, 251.15025; found, 251.14924.

FT-IR (ῦ in cm^-1^): 3349 (w), 3310 (m), 2934 (m), 2866 (w), 1632 (m), 1610 (s), 1548 (s), 1501 (s), 1447 (m), 1351 (w), 1289 (m), 1195 (w), 1122 (w), 1018 (w), 938 (s), 870 (m), 839 (m), 728 (m).

MP (DSC): Tm = 182°C

**
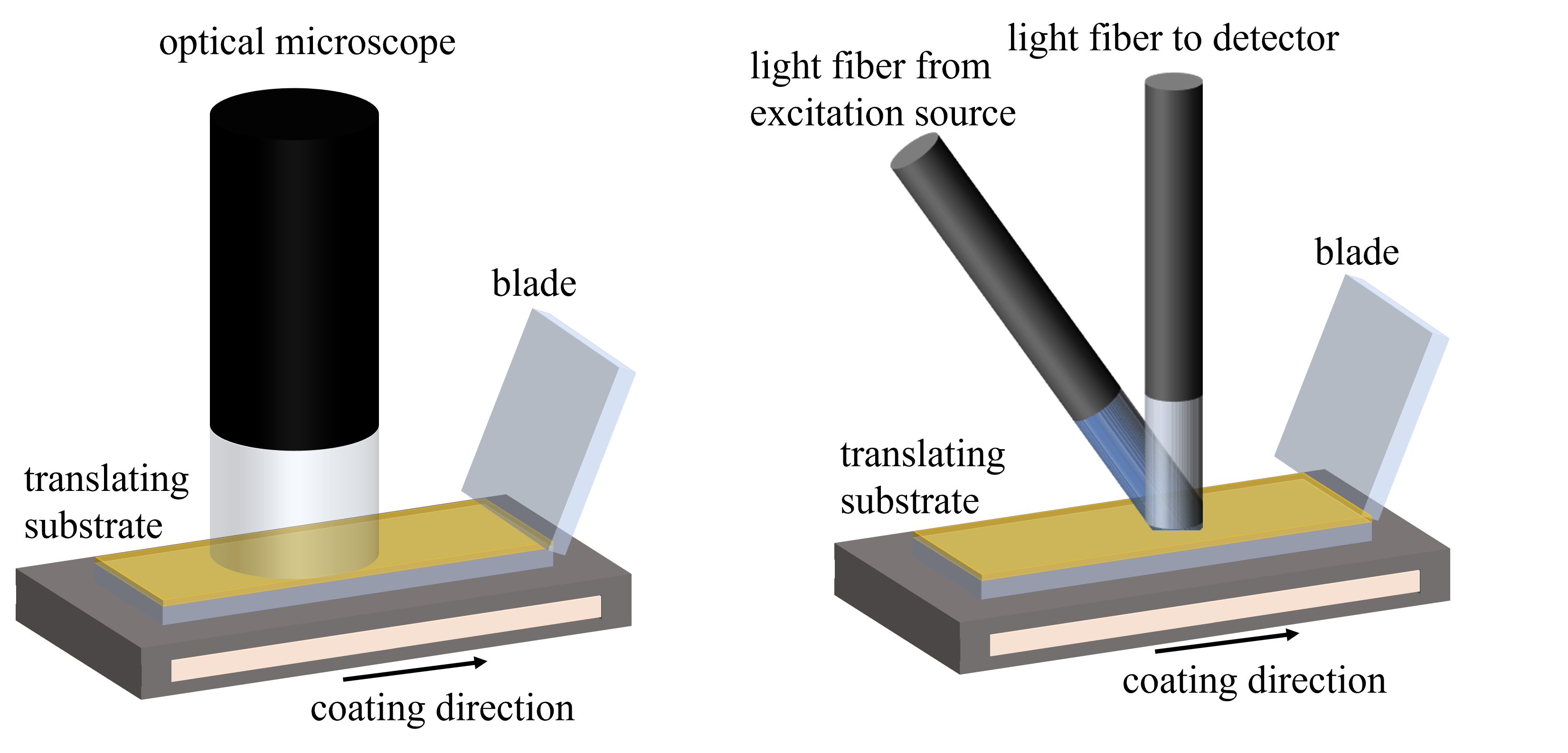
**

**Figure S1:** Schematic drawing of setup used for measuring the time-resolved optical microscopy in reflection mode (left) and time-resolved spectroscopy (right).

**S2: In-depth evaluation of time-resolved optical microscopy studies**

Detailed investigations of the time-resolved optical microscopy images reveal the formation process of the intermediate states. Tracking the size of a single growing structure along its long axis over the course of multiple images allows to extract a growth rate in micrometers per second. We performed multiple such evaluations per sample to estimate the average growth rate of a single structure for different additive concentrations along their long axis. An exemplary evolution is shown below:


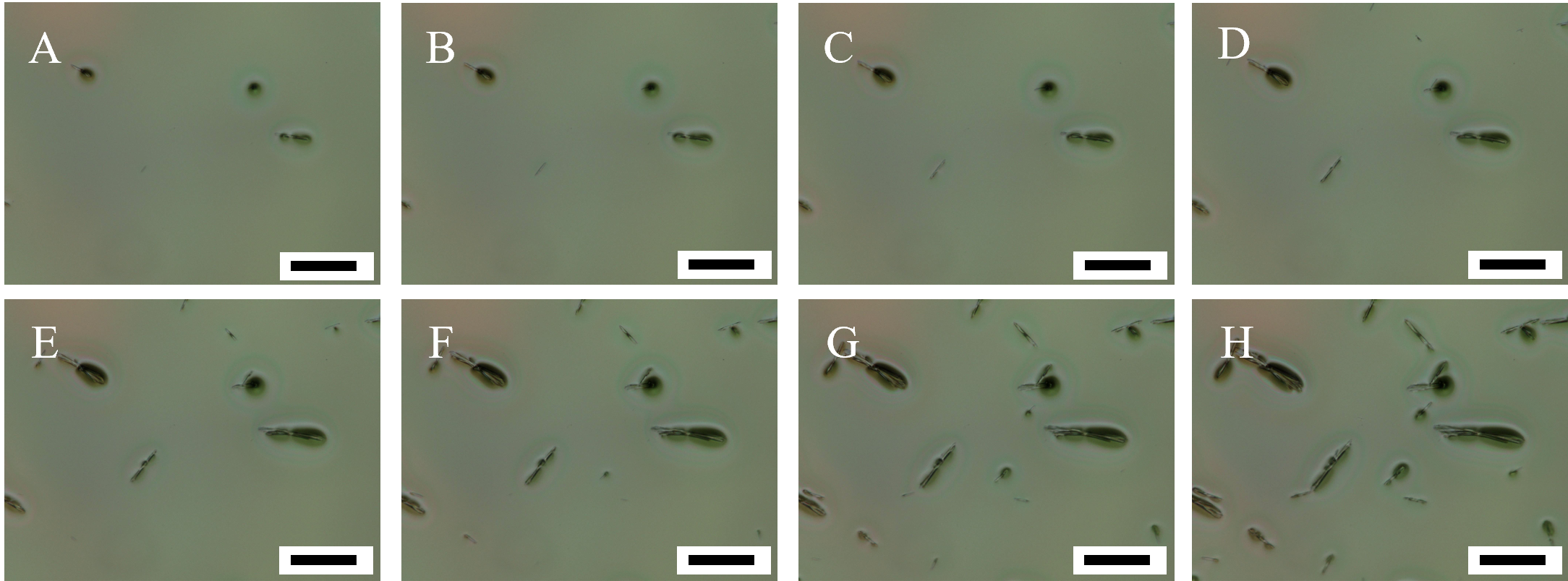


**Figure S2:** Time-resolved optical microscopy images during the formation of intermediate states in neat MAPbI_3_ solution in DMF. Small structures are formed, which quickly grow, especially along their long axis. Each shown image is 0.2 s apart, while the microcopy images were taken with a rate of 15 fps. The scale bar is 100 µm.


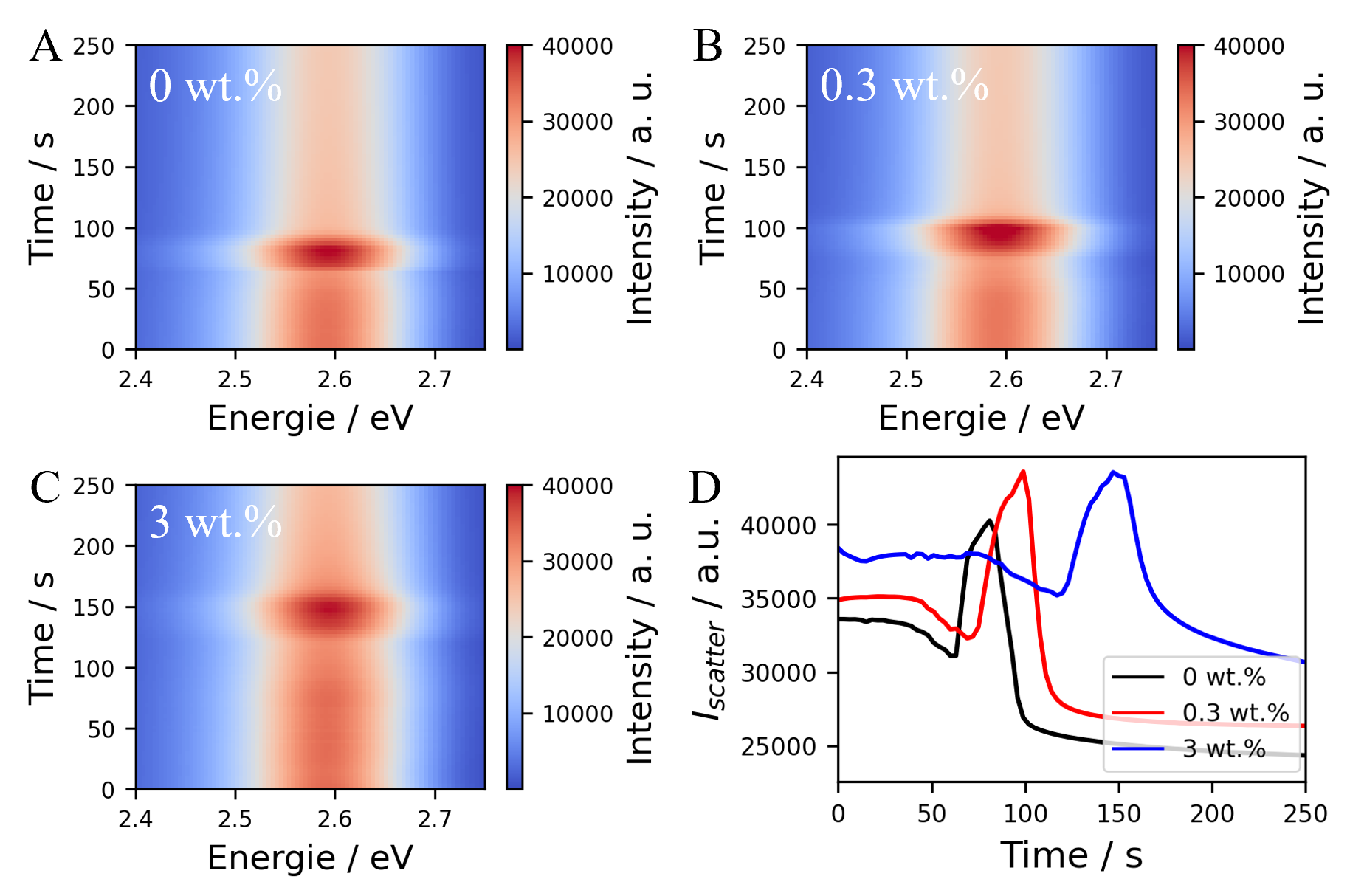
**Table S1:** Averaged growth rates of the structures during the intermediate state formation from a MAPbI_3_ solution in DMF with different amounts of SBBA additive.

| **Sample** | **0 wt.%** | **0.3 wt%** | **3 wt%** |
| --- | --- | --- | --- |
| **Growth Rate** | (60 ± 3) µm/s | (62 ± 8) µm/s | (20 ± 1) µm/s |

**Figure S3:** (A)-(C) Intensity maps of scattered light during the film drying for the samples with 0 wt.%, 0.3 wt.% and 3 wt.%. (D) Time evolution of the intensity of the scattered light for the three different samples with different contents of SBBA. The intensity is extracted with a line cut at 2.59 eV.

**S3: Fitting of photoluminescence spectra and calculation of coherence length**

We corrected the raw PL spectra with associated dark counts and applied a Savitzky–Golay filter as shown in Figure S4. In addition, the measured PL data features a broad signal around 2.59 eV due to the scattering of the incident light at the surface. This broad background signal has a tail up to 1.7 eV (730 nm). The contribution of this tail can be fitted with a Lorentz function and evolves with time and needs to be subtracted for each spectrum. For further analysis, we fit each corrected spectrum with seven hyperbolic secants ^[1-4]^ with a fixed position and width to describe the PL distribution. The seven functions were chosen to cover the full width of the PL signal. An exemplary fit is shown in Figure S5. The position of the peaks can be used to estimate the size of MAPbI_3_ crystals using $E_{PL}\left( d \right)=E_{bulk}+\frac{b}{d^{2}}$, thereby the energy of the PL peak can be used to estimate the crystallite size d using the PL position of bulk MAPbI_3_ and a constant b.^[3,5,6]^ In accordance with previous studies, we choose a *b* value of 3 eVnm^2^ and a bulk PL peak energy of 1.59 eV.^[3,6]^ From the intensity of each peak we calculate the fraction of each confinement size distribution as shown in Figure S6.


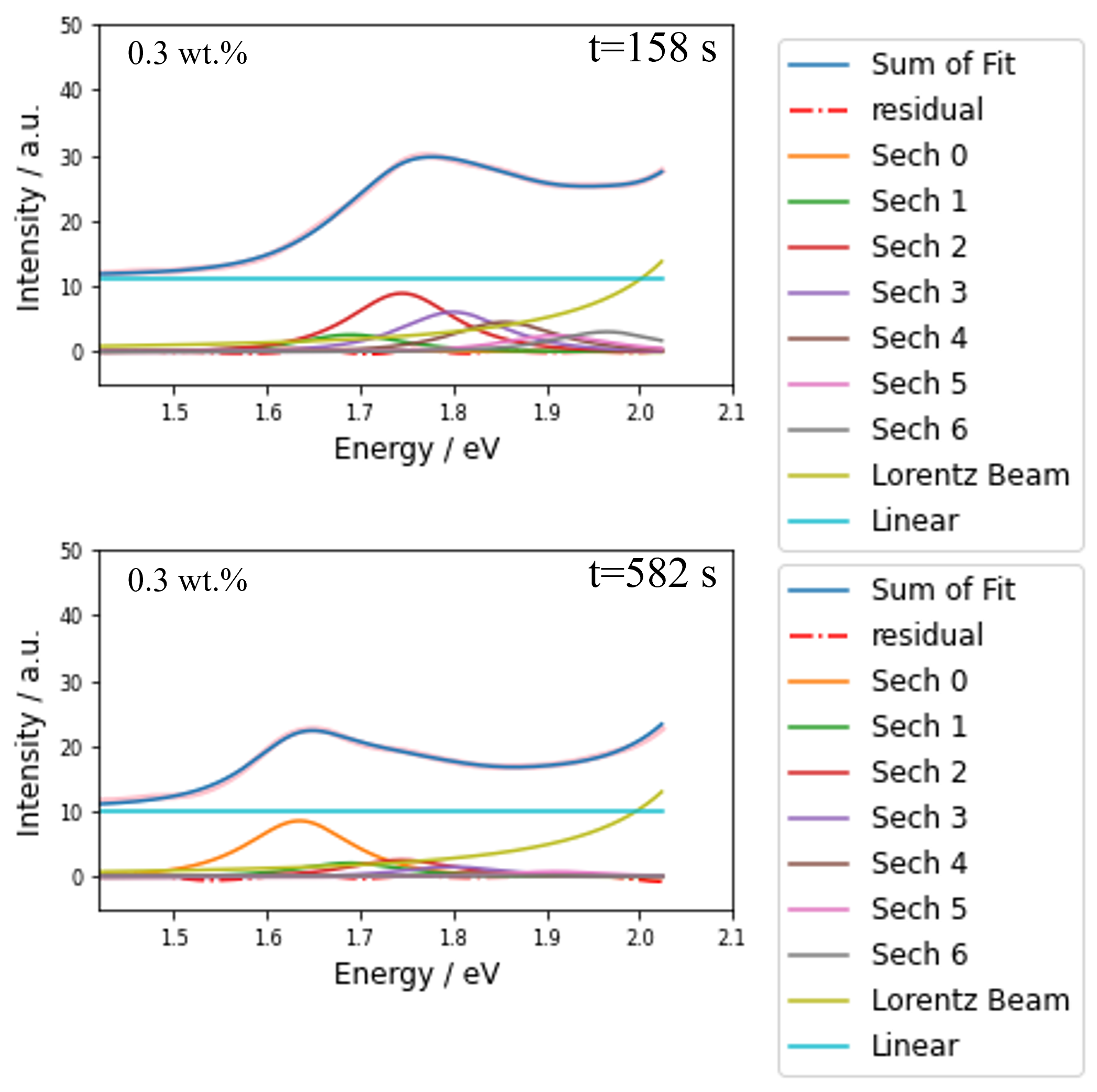

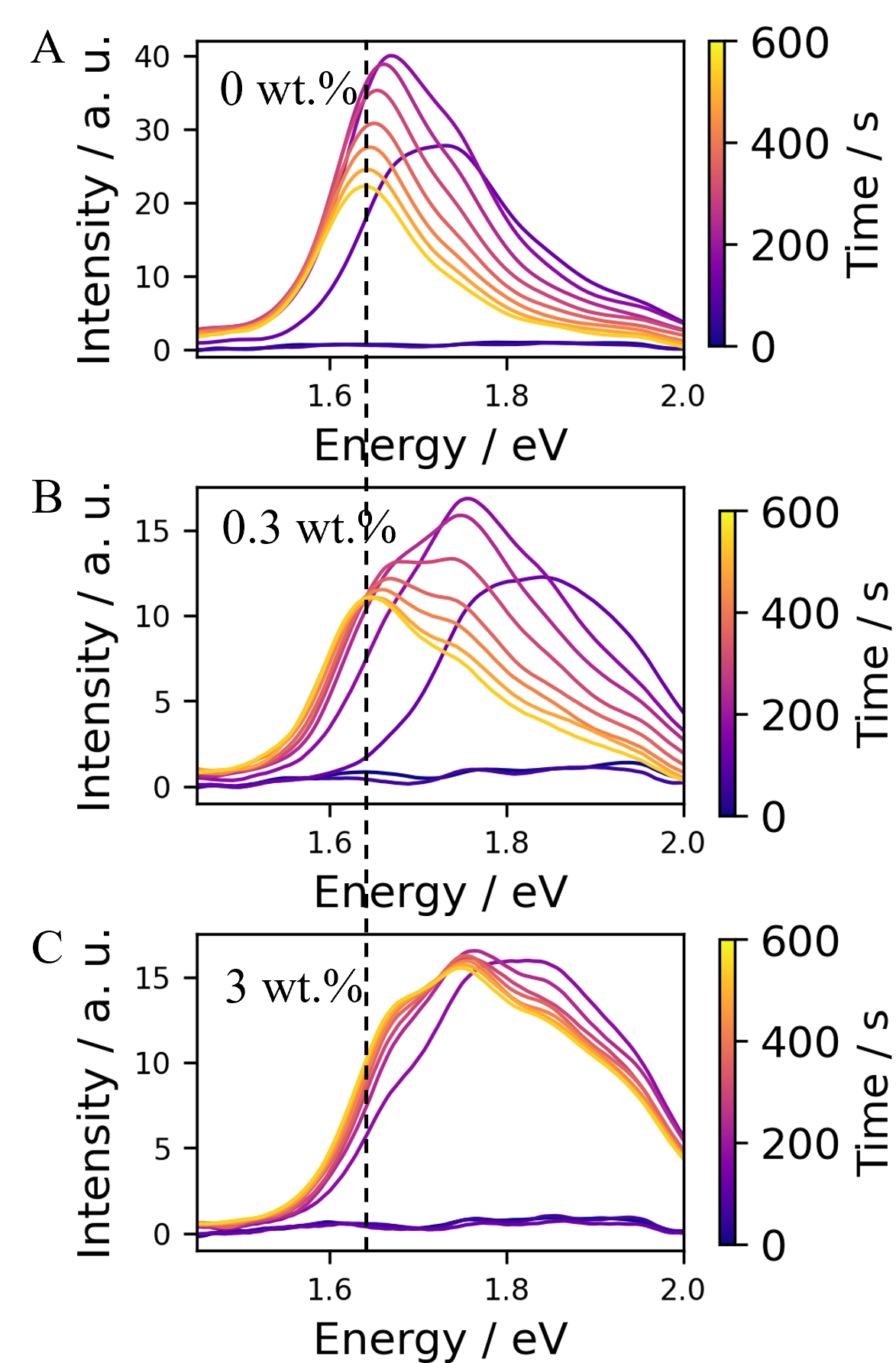
**Figure S4:** Complementary representation of Figure 3 in the main text. PL spectra at different times after blade coating for the samples with 0 wt.%, 0.3 wt.% and 3 wt.% SBBA. The dashed line indicates the final peak position for the thin film without SBBA.

**Figure S5:** PL spectra of MAPbI_3_ thin film with 0.3 wt.% of SBBA at two different times ((A) 156 s, (B) 582 s). Shown together with individual parts of the fit and the final fit (blue line).


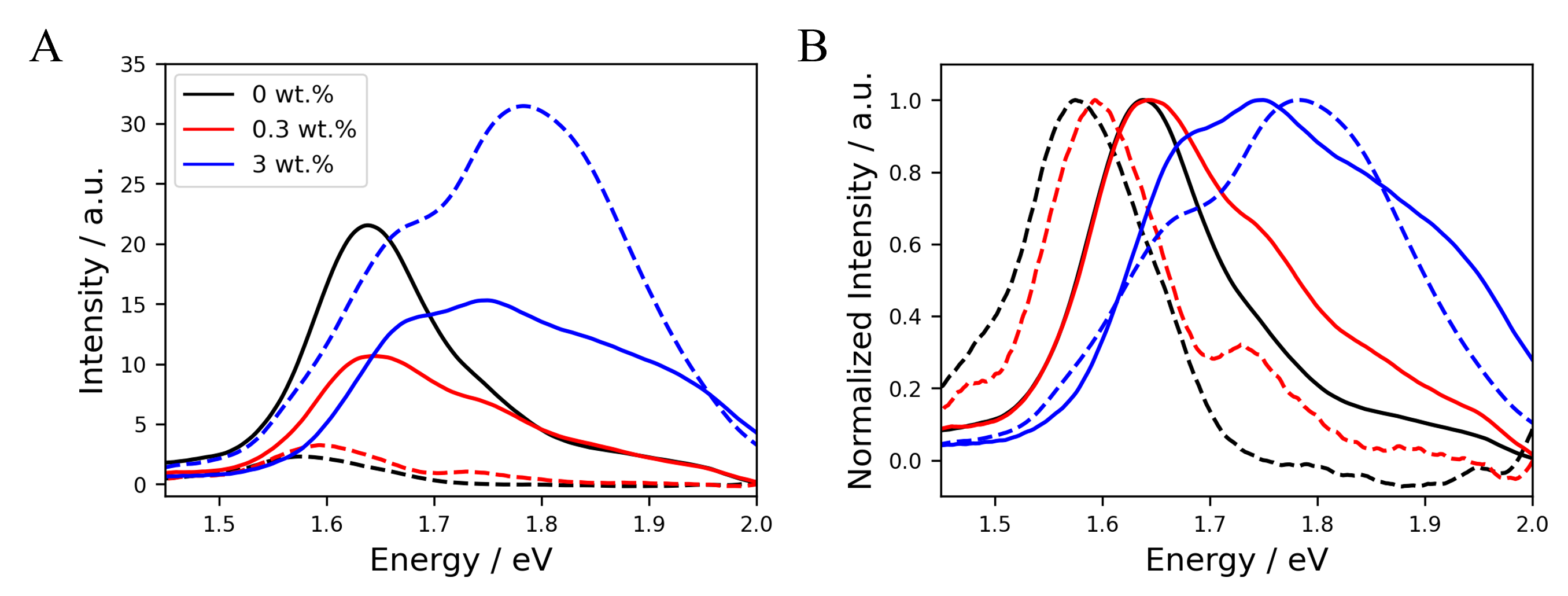

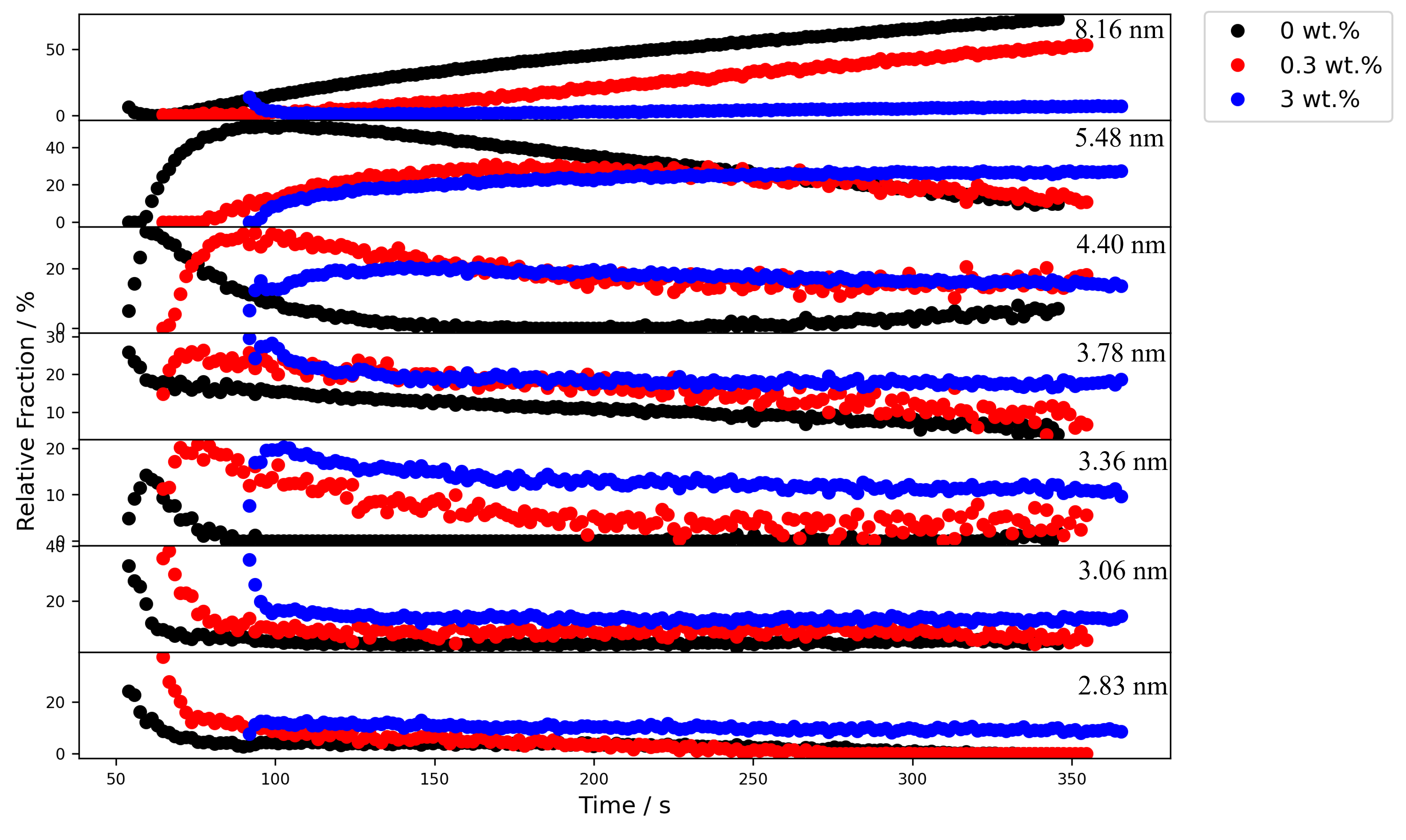
**Figure S6:** Time evolution of the fraction of each length distribution calculated from the fitted peak for the solution with 0 wt.% (black), 0.3 wt.% (red) and 3 wt.% (blue). The values on the right show the calculated confinement size for each hyperbolic secant. The exact value of the confinement size may differ depending on the used *b* value during the calculation.

**Figure S7:** Measured (A) and normalized (B) PL spectra before (solid lines) and after (dashed lines) thermal annealing of the films at 100 °C for 10 min.

**S4: Preparation of reference samples**

To evaluate the function of the SBBA during the structure formation process, three different reference compounds (Ref 1: butylamine, Ref 2: phenylethylamine, Ref 3: hexane-1,6-diamine) with amine end groups, but without hydrogen bond forming amides, which are a central part of the SBBA structure, are examined (see Figure S8). The solutions for the MAPbI_3_ thin film preparation were prepared in the same way as described in SI S2. All solutions were prepared with 1 wt.% of the reference compound and compared to a sample with 1 wt.% of SBBA. The crystallization process was examined with time-resolved light scattering and PL, as shown in Figure S9-12.

**
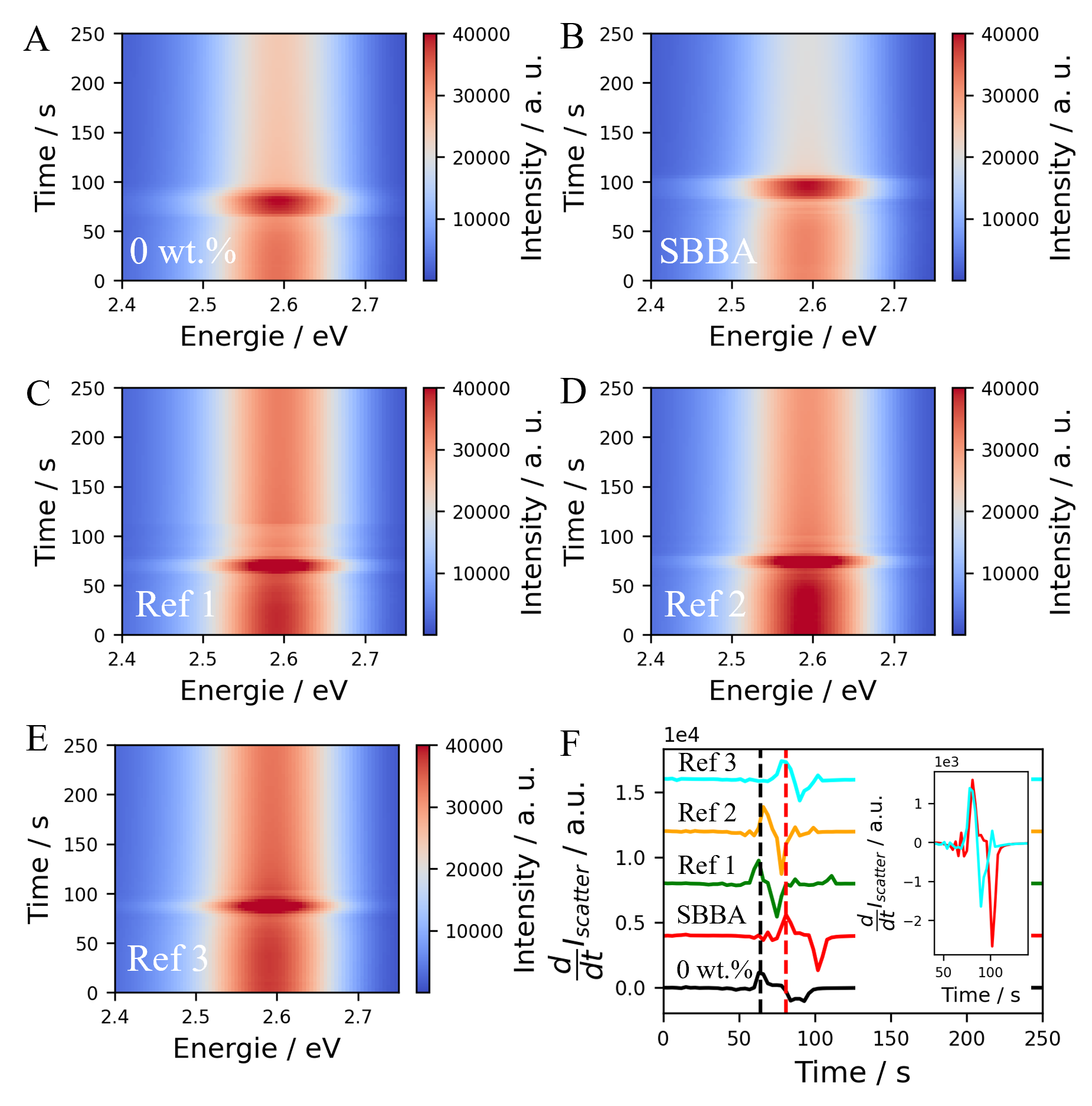
Figure S8:** (A)-(E) Intensity maps of scattered light signal during the film drying for the samples with 0 wt.%, 1 wt.% samples with the following additives: SBBA, Ref 1 (butylamine), Ref 2 (phenylethylamine), Ref 3 (hexane-1,6-diamine) as labelled. (F) Time derivative of the intensity of the scattered light signal for the different samples. The intensity was extracted with a line cut at 2.59 eV. The dashed lines indicate the peak maximum of the samples with 0 wt.% (black) and 1 wt.% (red) of SBBA. The inset shows the time derivative of the light scattering signal for the SBBA and Ref 3 (hexane-1,6-diamine) in comparison. For both additives a delay in intermediate phase formation is observed, while the broadening can only be observed for the SBBA.


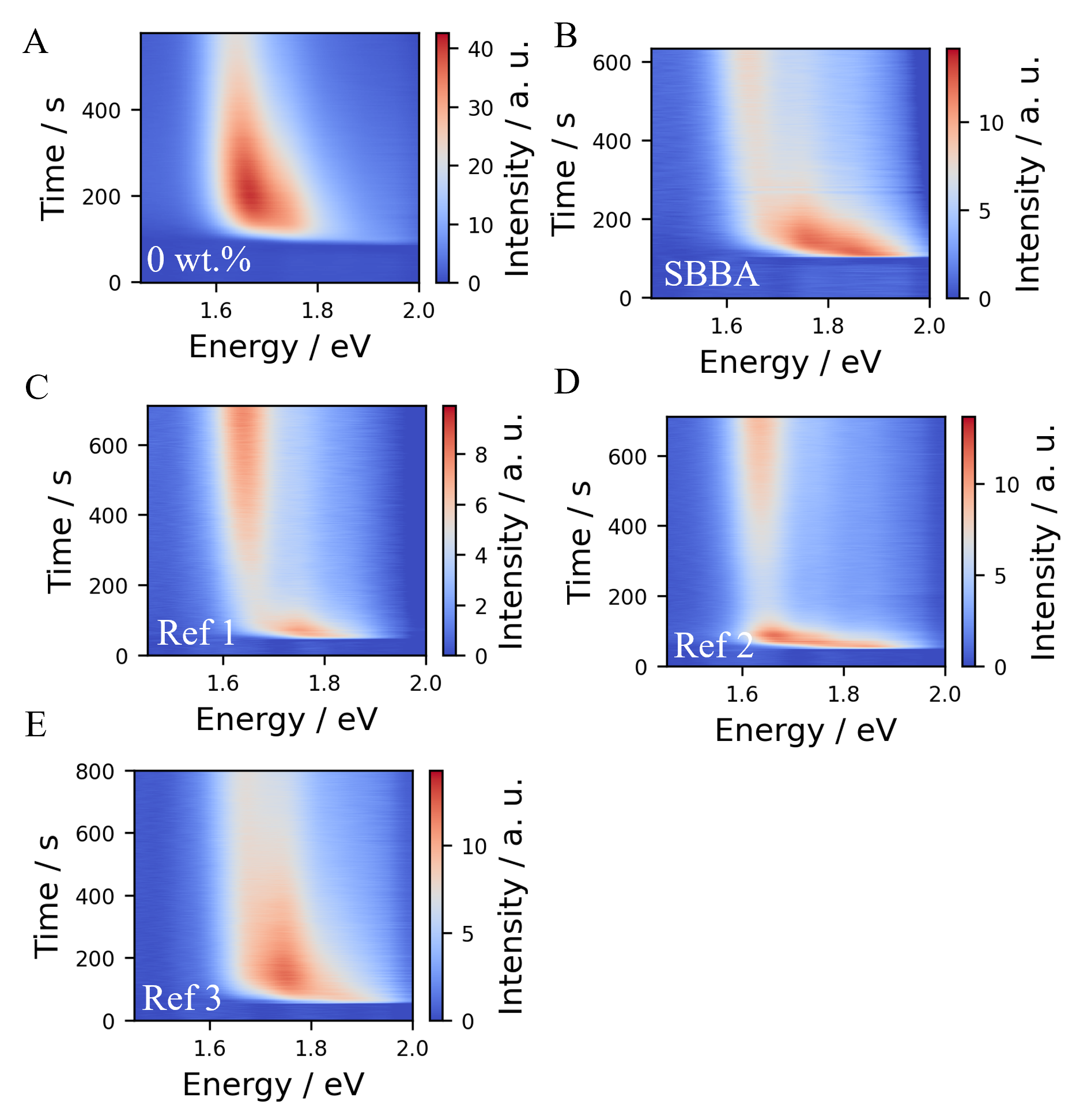
**Figure S9:** (A)-(E) Intensity maps as a function of time for the PL signal showing the conversion into the perovskite phase. Comparison of the neat sample with samples with 1 wt.% of SBBA and with 1 wt.% of the reference compounds (Ref 1- Ref 3) as indicated.


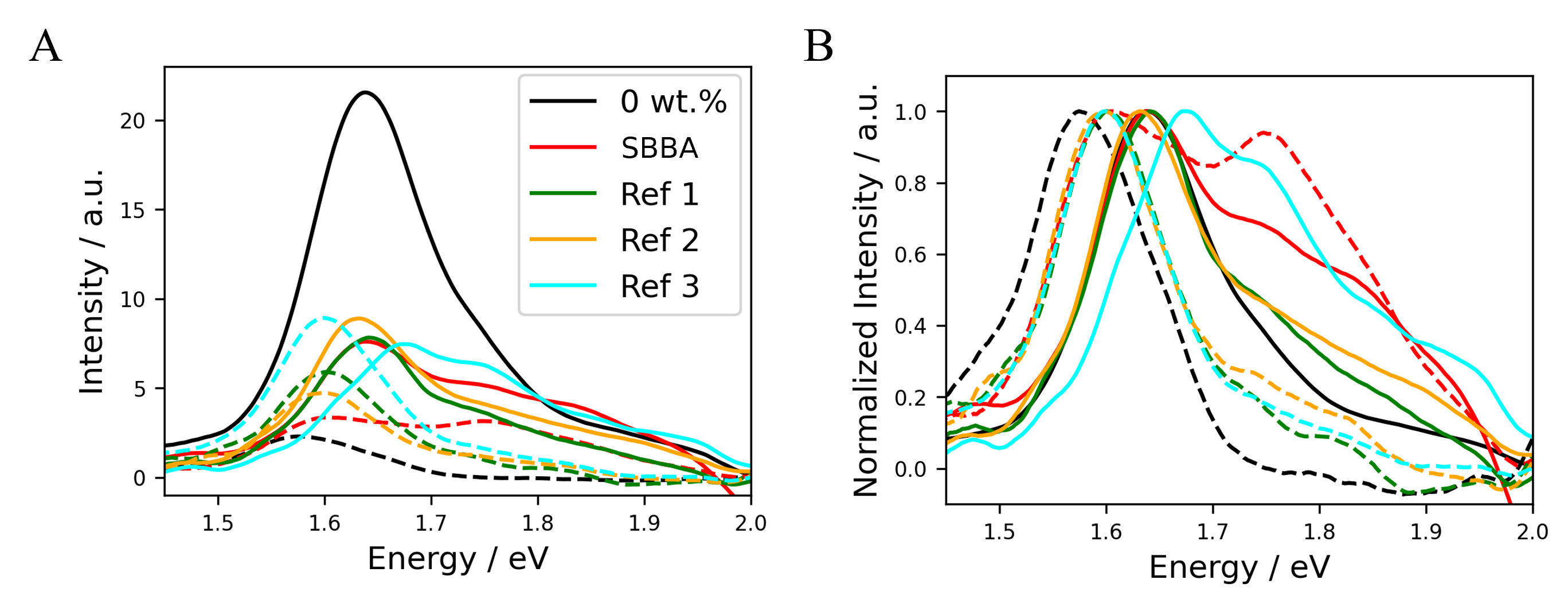
**Figure S10:** (A)-(E) Complementary representation of Figure S9. PL spectra at different times after starting the blade coating for the samples with different additives as indicated. (F) Calculated resulting fraction
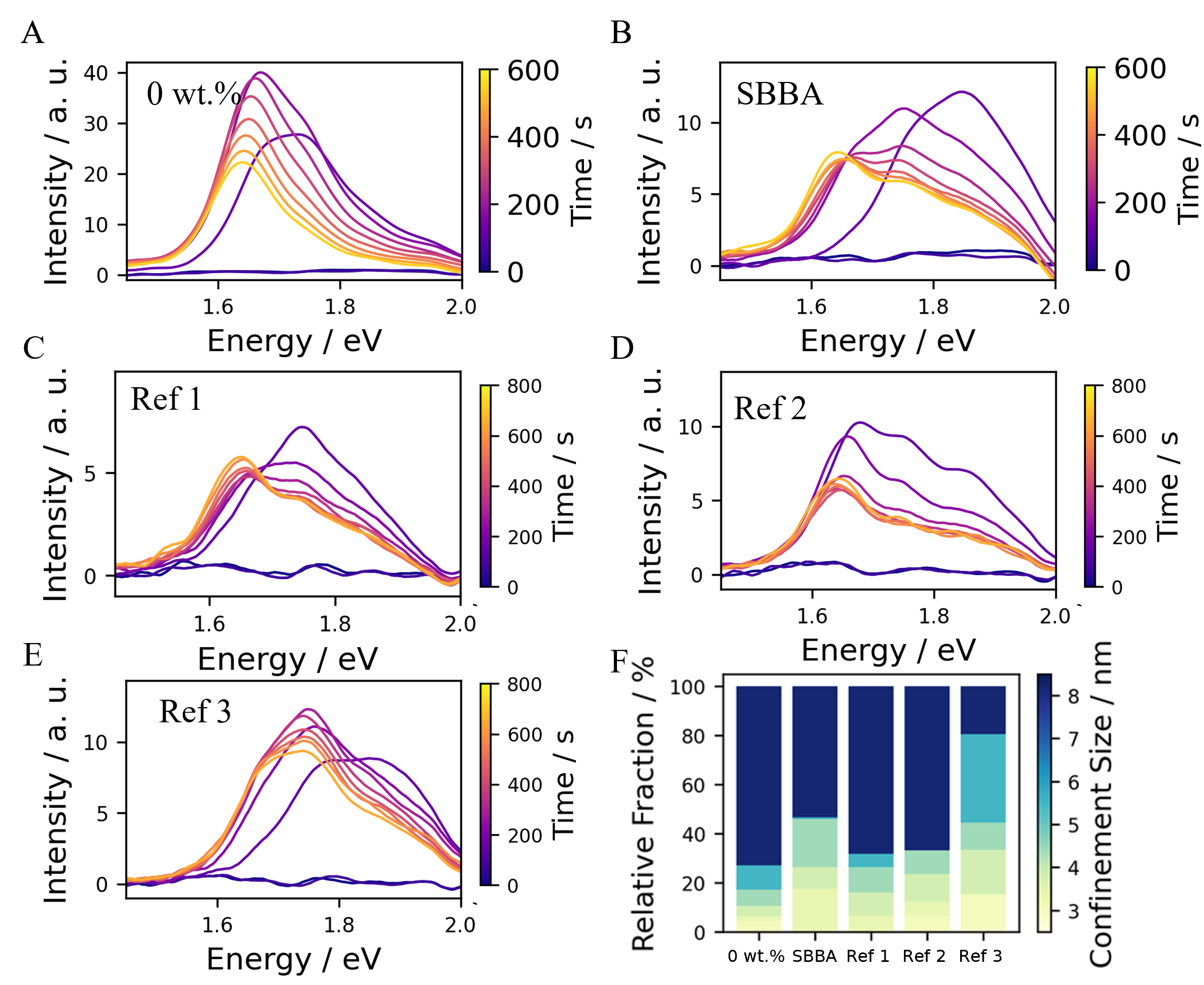
of the different crystallite sizes for the final films before the thermal annealing obtained from the fits of the PL signals of the dried, pre-annealed film.

**Figure S11:** Measured (A) and normalized (B) PL spectra before (solid line) and after the thermal annealing (dashed line) of the films at 100 °C.

**S5: Assignment of the additional peaks in the WAXS measurement**


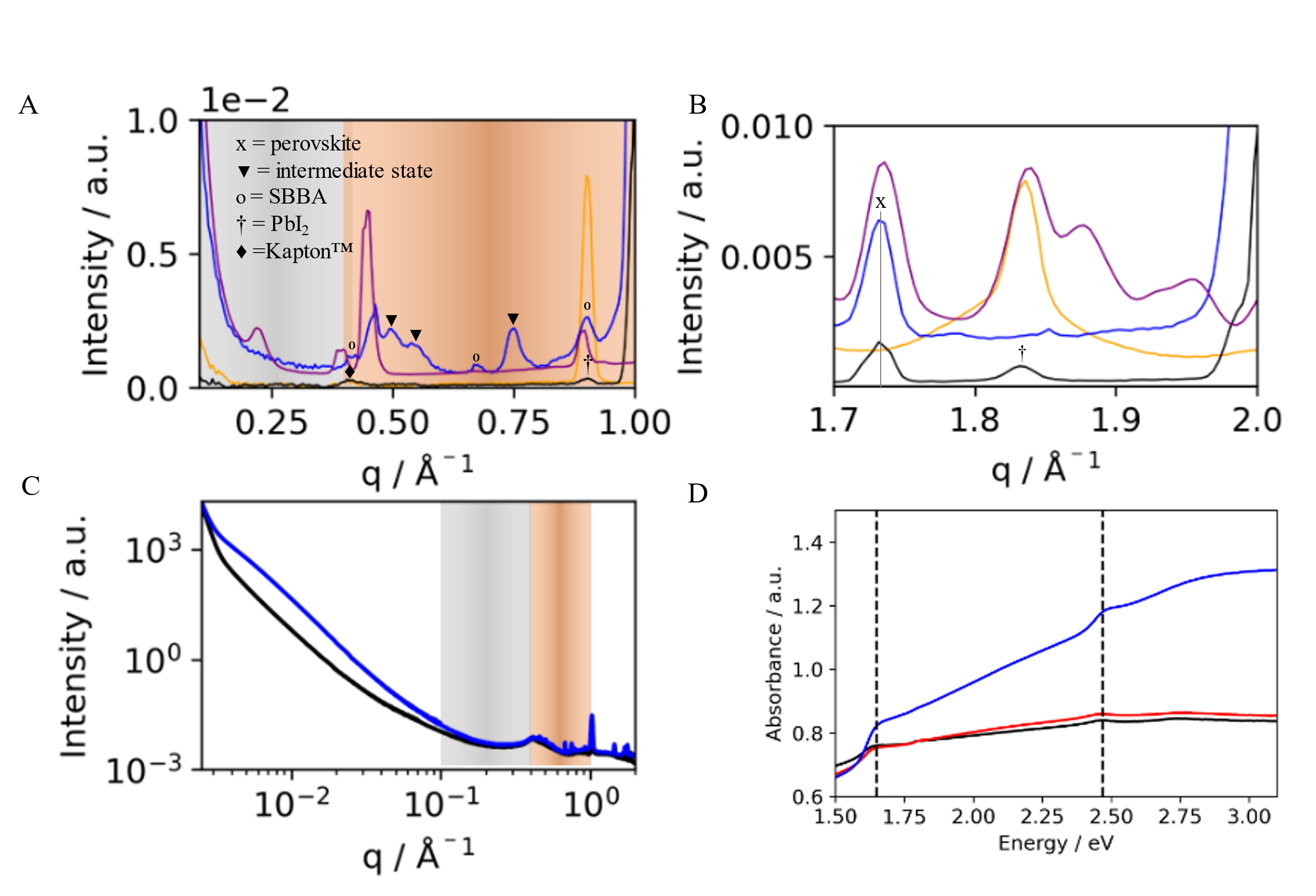
To assign the additional peaks that occurred in the WAXS measurement of the MAPbI_3_ sample with SBBA, we carried out WAXS measurements on neat SBBA and PbI_2_ powder. The PbI_2_ was also measured between two Kapton™ foils, and the SBBA was measured in a capillary. The 2D WAXS data was radially averaged to obtain scattering intensity vs q. The extracted values for the additional peaks are shown in Table S2. The peaks at 0.41 Å^-1^, 0.46 Å^-1^, 0.67 Å^-1^ are assigned to the SBBA. The peaks at 0.49 Å^-1^, 0.54 Å^-1^ and 0.75 Å^-1^ are attributed to the (MA)_2_(DMF)_2_Pb_2_I_6_ intermediate state, which is formed during the drying of the thin film.^[7]^ At 0.9 Å^-1^, a peak is visible in the sample with neat MAPbI_3_ and MAPbI_3_ with SBBA; at the same position, a peak can be detected in the neat SBBA and the PbI_2_. If the peak results from PbI_2_, we expect to see the second order of the peak. This is observed for the sample without additive, where the first and second order of the PbI_2_ peak are clearly visible. For the sample with SBBA, no 2^nd^ order of this peak is visible in the sample with SBBA, thus we can assign this peak to the SBBA.

**Figure S12:** (A), (B) Radially averaged WAXS data for different q-ranges. The additional peaks could be assigned using the spectra of the neat SBBA (circle) and literature values (solid triangle). The colored ranges indicate the typical ranges for 2D perovskite signals (grey) and intermediate phases (orange). (C) Extended q-range to the SAXS region for measured samples. (D) Absorbance spectra of annealed thin films.

| **Peak value** | **Assignment** | **Reference value** |
| --- | --- | --- |
| 0.410±0.010 Å^-1^ | SBBA | 0.39 Å^-1 *^ |
| 0.460±0.005 Å^-1^ | SBBA / intermediate | 0.45 Å^-1 *^ / 0.46 Å^-1 Ref:[7]^ |
| 0.490±0.010 Å^-1^ | intermediate | 0.5 Å^-1 Ref:[7]^ |
| 0.550±0.005 Å^-1^ | intermediate | 0.58 Å^-1 Ref:[7]^ |
| 0.680±0.005 Å^-1^ | SBBA | 0.67 Å^-1 *^ |
| 0.750±0.005 Å^-1^ | intermediate | 0.73 Å^-1^ ^Ref:[7]^ |
| 0.900±0.005 Å^-1^ | SBBA | 0.89 Å^-1 *^ |

**Table S2:** Peak values of the additional peaks in the WAXS measurement of the sample with SBBA and their assignment. The reference values marked with * were obtained by the measurement of the neat SBBA material.


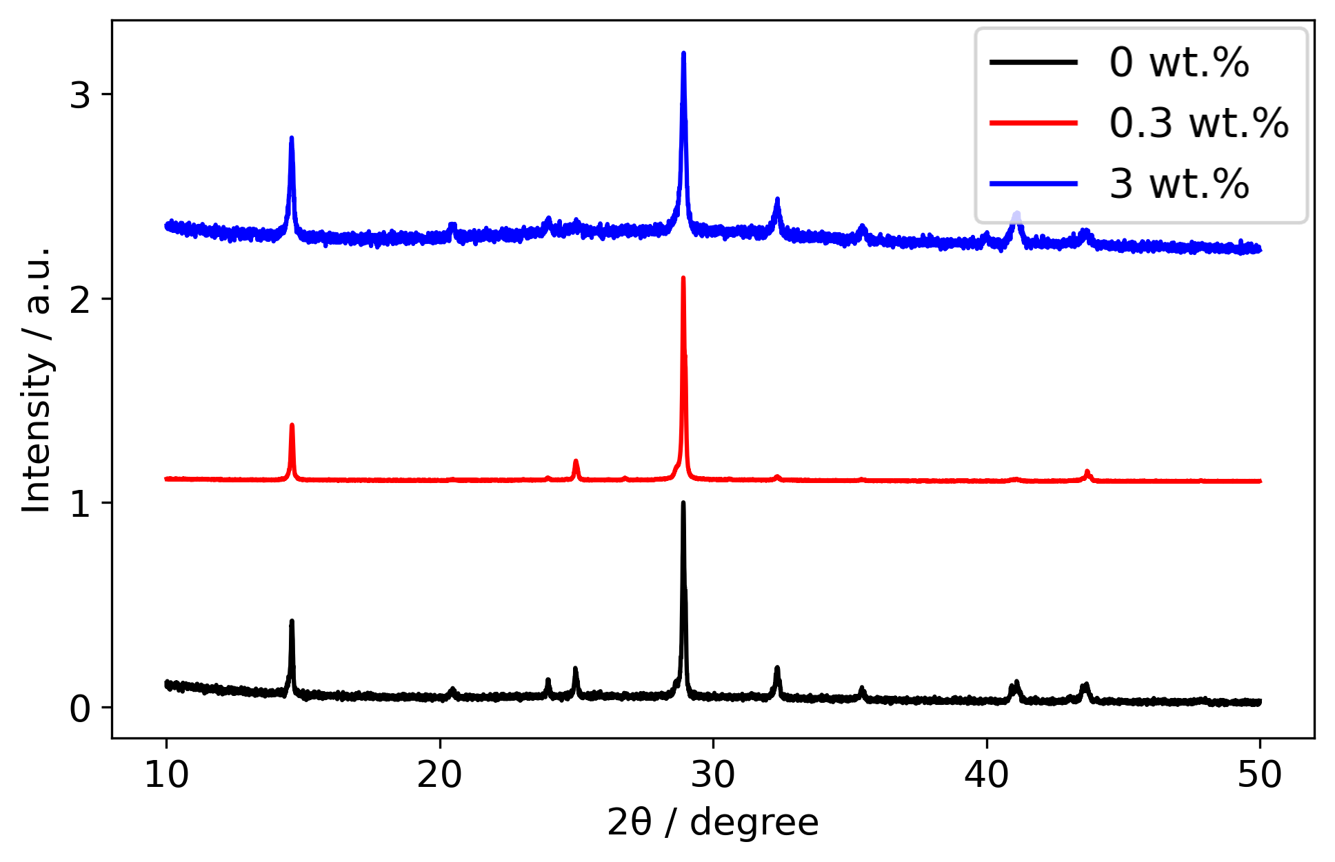


**Figure S13:** XRD measurements of MAPbI_3_ films with different content of SBBA. Curves are normalized and are linearly shifted for clarity.


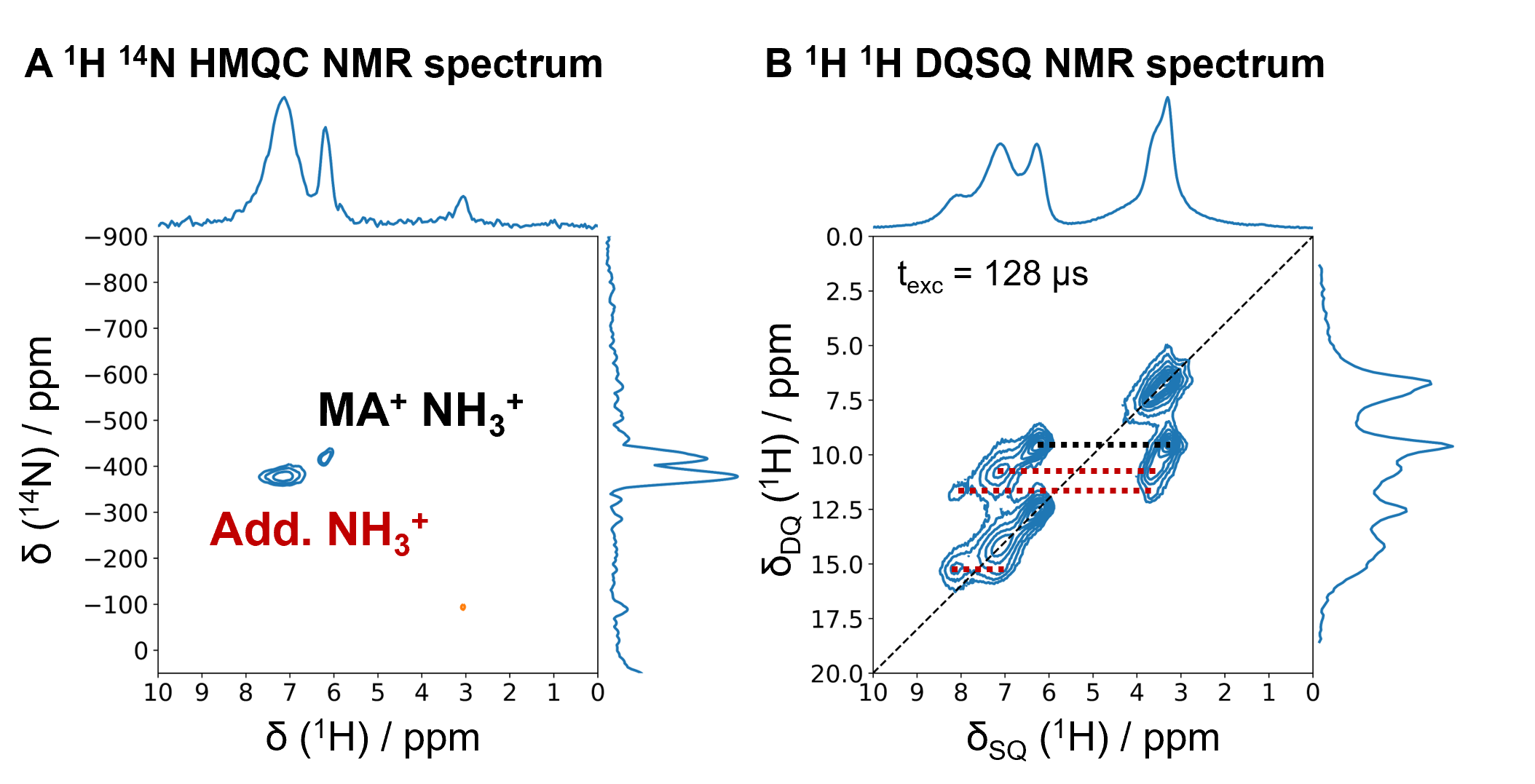


**Figure S14:** A) ^1^H ^14^N HMQC and B) ^1^H ^1^H DQSQ NMR spectra of 3 wt.% SBBA in MAPbI_3_. B) The cross correlations (off-diagonal) signals between ^1^H species of the additive are highlighted by red dotted lines, and the MA-MA correlations within the perovskite is marked by the black dotted line.


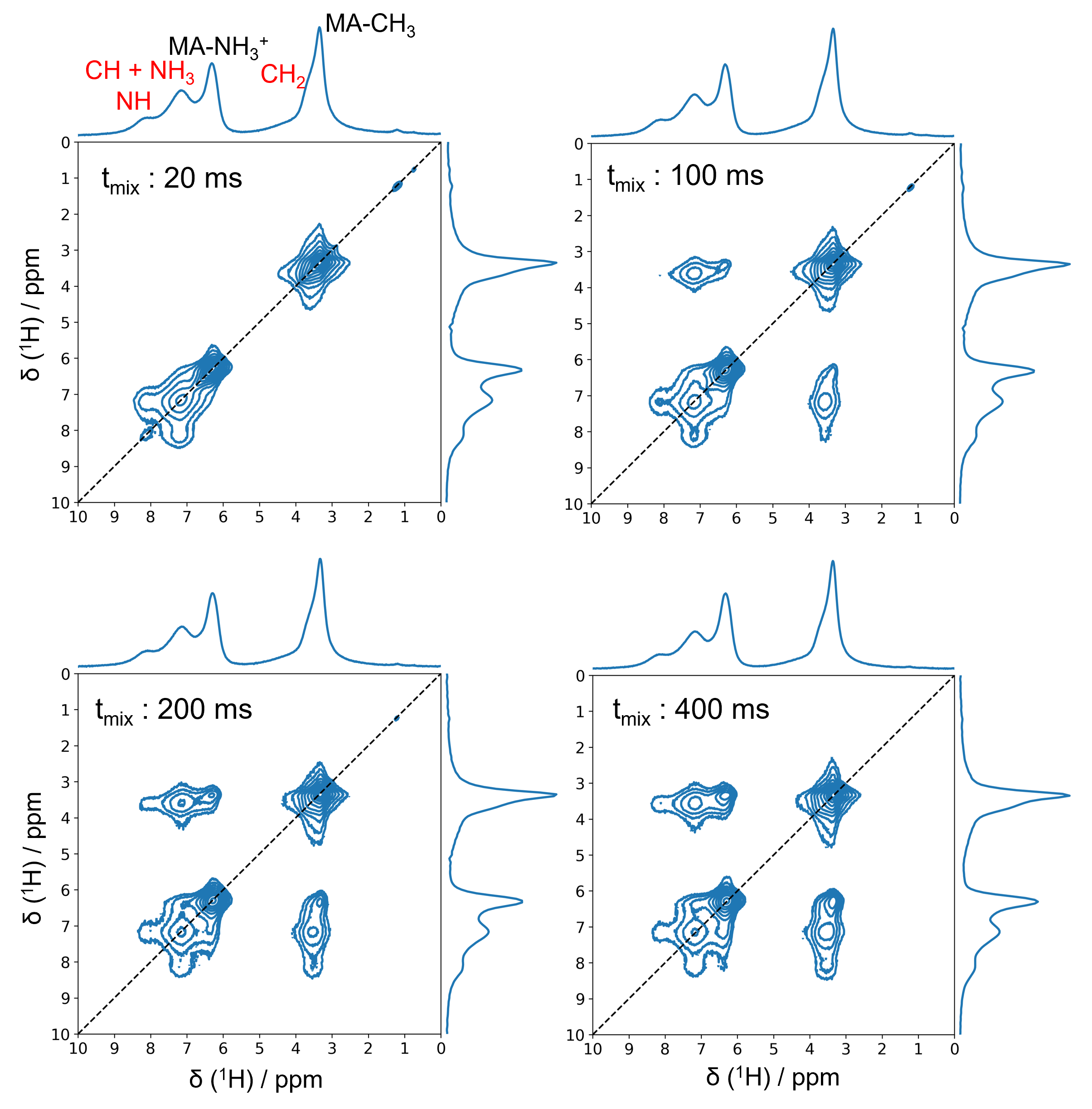


**Figure S15:** 2D ^1^H ^1^H EXSY NMR spectrum of MAPbI_3_ with 3 wt.% SBBA for additional mixing times.


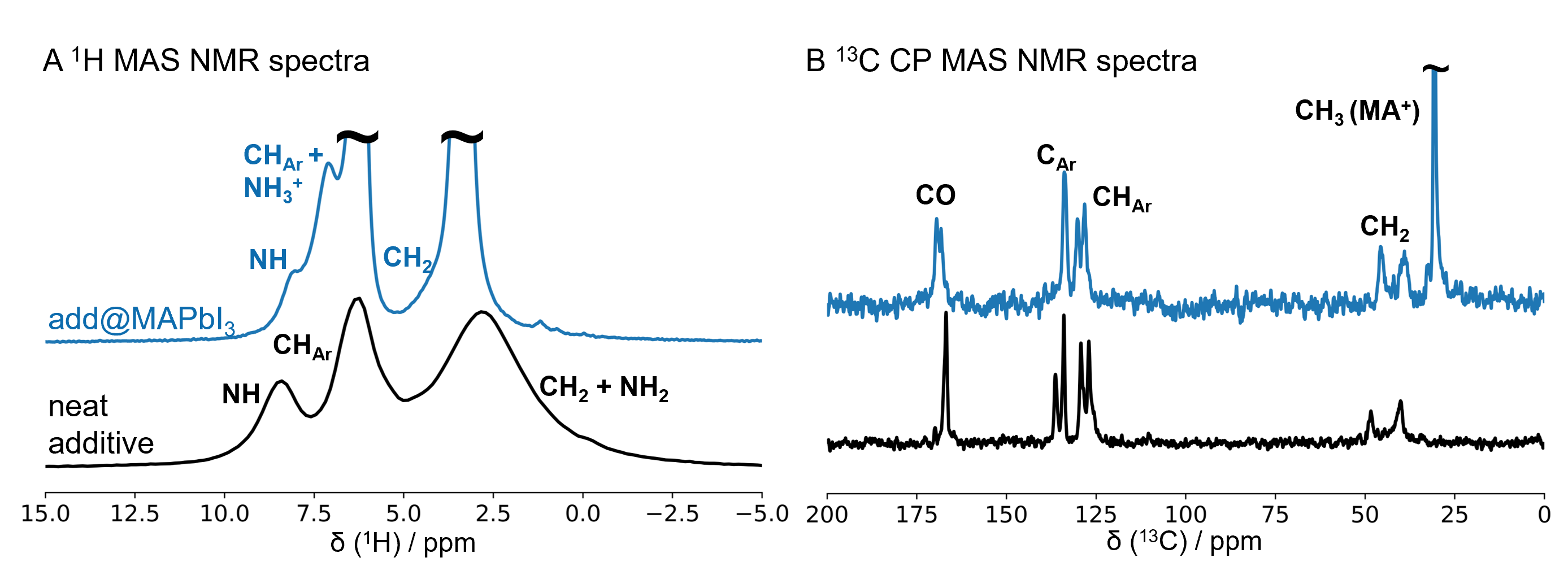


**Figure S16:** (A) Solid-state ^1^H MAS (left) and(A) ^13^C CP MAS NMR spectra of 3 wt.% SBBA in MAPbI_3_ (blue) and neat additive (black). (B) The assignment of the signals is indicated by the abbreviation of the corresponding ^1^H species (see main text for details).

| **Assignment** | **Chemical shift / ppm** | **Norm. Integral** | **FWHM /Hz** | **G/L ratio** |
| --- | --- | --- | --- | --- |
| CH_3_ (MA^+^) | 3.29 | 20.4 | 179 | 0.18 |
| NH_3_^+^ (MA^+^) | 6.25 | 19.3 | 267 | 0.50 |
| CH_2_ (SBBA) | 3.59 | 8.4 | 405 | 0.00 |
| CH_Ar_ + NH_3_^+^ (SBBA) | 7.13 | 10.3 | 709 | 0.60 |
| NH (SBBA) | 8.15 | 2.0 | 477 | 0.81 |

**Table S3**. Parameters and normalized integrals form deconvoluting the quantitative ^1^H MAS NMR spectrum of MAPbI_3_ with 3 wt.% SBBA additive (Fig. 6a, main text) and the assignment of the observed ^1^H signals.

| **Assignment** | **Chemical shift / ppm** | **Norm. Integral** | **FWHM /Hz** | **G/L ratio** |
| --- | --- | --- | --- | --- |
| CH_2_ + NH_2_ | 2.75 | 12.2 | 2176 | 0.46 |
| CH_Ar_ | 6.30 | 4.3 | 902 | 0.58 |
| NH | 8.45 | 2.0 | 850 | 0.49 |

**Table S4**. Parameters and normalized integrals form deconvoluting the quantitative ^1^H MAS NMR spectrum of the neat SBBA additive (Fig. S16a) and the assignment of the observed ^1^H signals.


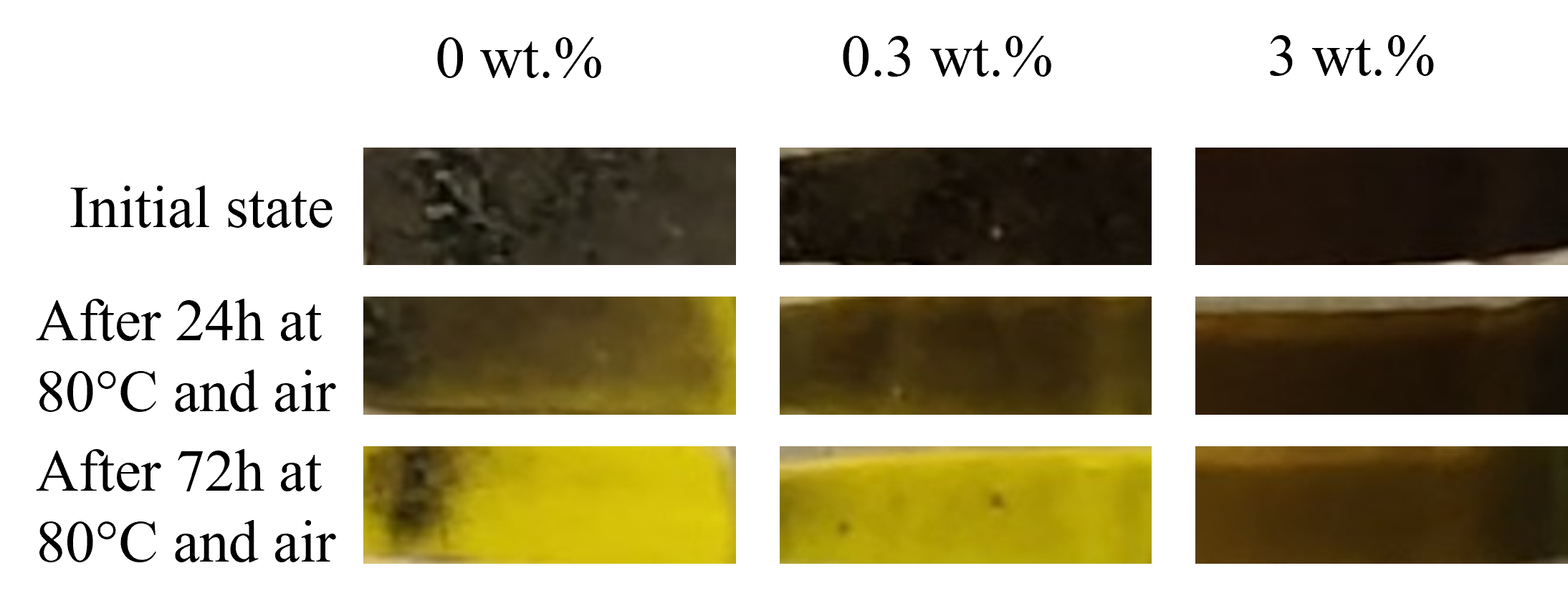
**Figure S17:** Photographs of thin films used for the thermally accelerated ageing study. The first row corresponds to the fresh thin film after thermal annealing at 100 °C, the second row corresponds to the thin film after ageing for 24 hours at 80°C in air at 35% humidity and the last row corresponds to the thin film after 72 hours of ageing at that condition. The photographed samples have a size of about 0.3 × 1 cm².

**Figure S18:** (A)-(D) 2D GIWAXS images of the initial state (after temperature annealing at 100 °C) and final state (after thermal treatment at 200 °C) of the samples as labelled. (E) Cake cuts along q_z_ for before (solid line, initial state) and after (dashed line, final state) the accelerated thermal ageing procedure of the film in nitrogen with 0 wt.% and 3 wt.% of SBBA. Each thin film was heated to 200 °C and held at that
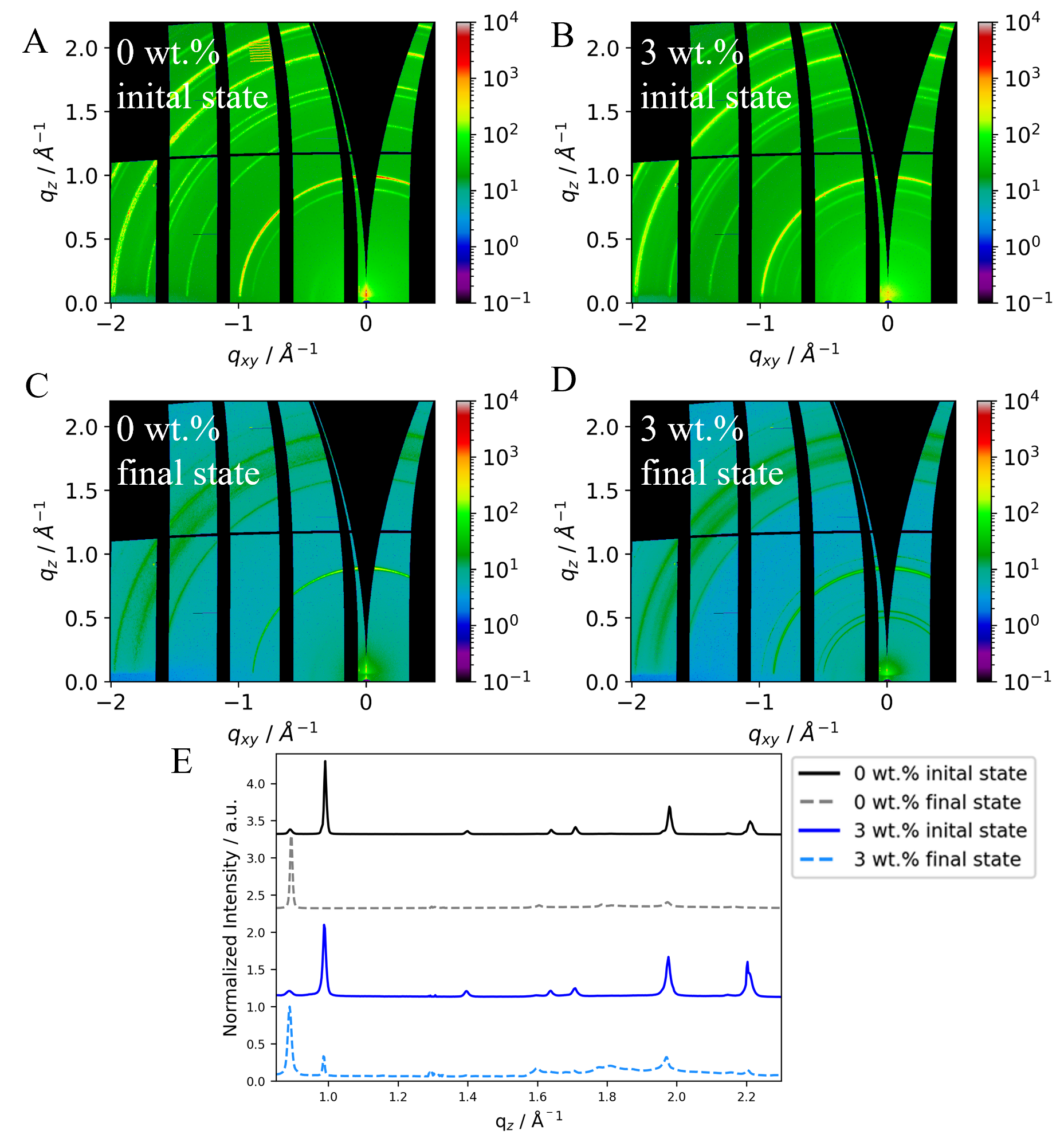
temperature for 5 min during the treatment. The shown data were taken prior and after the accelerated thermal ageing procedure at room temperature.

**Supplementary references**

1. S.-G. Kim, J.-H. Kim, P. Ramming, Y. Zhong, K. Schötz, S. J. Kwon, S. Huettner, F. Panzer, N.-G. Park, *Nat. Commun.* **2021**, *12*, 1554.

2. K. Schötz, A. M. Askar, W. Peng, D. Seeberger, T. P. Gujar, M. Thelakkat, A. Köhler, S. Huettner, O. M. Bakr, K. Shankar, F. Panzer, *J. Mater. Chem. C* **2020,** *8*, 2289.

3. K. Schötz, C. Greve, A. Langen, H. Gorter, I. Dogan, Y. Galagan, A. J. J. M. Breemen, G. H. Gelinck, E. M. Herzig, F. Panzer, *Adv. Opt. Mater.* **2021,** *9*, 2101161.

4. K. Schötz, F. Panzer, *J. Phys. Chem. A* **2021,** *125*, 2209.

5. Y. Kayanuma, *Phys. Rev. B* **1988**, *38*, 9797.

6. S. Biberger, K. Schötz, P. Ramming, N. Leupold, R. Moos, A. Köhler, H. Grüninger, F. Panzer, *J. Mater. Chem. A* **2022,** *10*, 18038.

7. A. A. Petrov, I. P. Sokolova, N. A. Belich, G. S. Peters, P. V. Dorovatovskii, Y. V. Zubavichus, V. N. Khrustalev, A. V. Petrov, M. Grätzel, E. A. Goodilin, A. B. Tarasov, *J. Phys. Chem. C* **2017**, *121*, 20739.
